# Features of Recently Transmitted HIV-1 Clade C Viruses that Impact Antibody Recognition: Implications for Active and Passive Immunization

**DOI:** 10.1371/journal.ppat.1005742

**Published:** 2016-07-19

**Authors:** Cecilia Rademeyer, Bette Korber, Michael S. Seaman, Elena E. Giorgi, Ruwayhida Thebus, Alexander Robles, Daniel J. Sheward, Kshitij Wagh, Jetta Garrity, Brittany R. Carey, Hongmei Gao, Kelli M. Greene, Haili Tang, Gama P. Bandawe, Jinny C. Marais, Thabo E. Diphoko, Peter Hraber, Nancy Tumba, Penny L. Moore, Glenda E. Gray, James Kublin, M. Juliana McElrath, Marion Vermeulen, Keren Middelkoop, Linda-Gail Bekker, Michael Hoelscher, Leonard Maboko, Joseph Makhema, Merlin L. Robb, Salim Abdool Karim, Quarraisha Abdool Karim, Jerome H. Kim, Beatrice H. Hahn, Feng Gao, Ronald Swanstrom, Lynn Morris, David C. Montefiori, Carolyn Williamson

**Affiliations:** 1 Division of Medical Virology & Institute of Infectious Diseases and Molecular Medicine, University of Cape Town and National Health Laboratory Service (NHLS), Cape Town South Africa; 2 Los Alamos National Laboratory and New Mexico Consortium, Los Alamos, New Mexico, United States of America; 3 Beth Israel Deaconess Medical Center, Boston, Massachusetts, United States of America; 4 Department of Surgery, Duke University Medical Center, Durham, North Carolina, United States of America; 5 Botswana-Harvard AIDS Institute Partnership, Gaborone, Botswana; 6 National Institute for Communicable Diseases (NICD), NHLS & University of the Witwatersrand, Johannesburg, South Africa; 7 Centre for the AIDS Programme of Research in South Africa (CAPRISA), University of KwaZulu-Natal, Durban, South Africa; 8 Perinatal HIV Research Unit, Faculty of Health Sciences, University of the Witwatersrand, Johannesburg and South African Medical Research Council, Cape Town, South Africa; 9 Vaccine and Infectious Disease Division, Fred Hutchinson Cancer Research Center, Seattle, Washington, United States of America; 10 South African National Blood Service, Weltevreden Park, South Africa; 11 Desmond Tutu HIV Centre, Department of Medicine and Institute of Infectious Disease and Molecular Medicine, University of Cape Town (UCT), Cape Town, South Africa; 12 Department for Infectious Diseases & Tropical Medicine, Klinikum University of Munich, LMU and German Center for Infection Research (DZIF) partner site Munich, Munich, Germany; 13 NIMR-Mbeya Medical Research Center, Mbeya, Tanzania; 14 US Military HIV Research Program, Walter Reed Army Institute of Research, Silver Spring, Maryland, United States of America; 15 International Vaccine Institute, Seoul, Republic of Korea; 16 Perelman School of Medicine, University of Pennsylvania, Philadelphia, Pennsylvania, United States of America; 17 Department of Biochemistry and Biophysics, University of North Carolina at Chapel Hill, Chapel Hill, North Carolina, United States of America; National Institutes of Health-NIAID, UNITED STATES

## Abstract

The development of biomedical interventions to reduce acquisition of HIV-1 infection remains a global priority, however their potential effectiveness is challenged by very high HIV-1 envelope diversity. Two large prophylactic trials in high incidence, clade C epidemic regions in southern Africa are imminent; passive administration of the monoclonal antibody VRC01, and active immunization with a clade C modified RV144-like vaccines. We have created a large representative panel of C clade viruses to enable assessment of antibody responses to vaccines and natural infection in Southern Africa, and we investigated the genotypic and neutralization properties of recently transmitted clade C viruses to determine how viral diversity impacted antibody recognition. We further explore the implications of these findings for the potential effectiveness of these trials. A panel of 200 HIV-1 Envelope pseudoviruses was constructed from clade C viruses collected within the first 100 days following infection. Viruses collected pre-seroconversion were significantly more resistant to serum neutralization compared to post-seroconversion viruses (p = 0.001). Over 13 years of the study as the epidemic matured, HIV-1 diversified (p = 0.0009) and became more neutralization resistant to monoclonal antibodies VRC01, PG9 and 4E10. When tested at therapeutic levels (10ug/ml), VRC01 only neutralized 80% of viruses in the panel, although it did exhibit potent neutralization activity against sensitive viruses (IC_50_ titres of 0.42 μg/ml). The Gp120 amino acid similarity between the clade C panel and candidate C-clade vaccine protein boosts (Ce1086 and TV1) was 77%, which is 8% more distant than between CRF01_AE viruses and the RV144 CRF01_AE immunogen. Furthermore, two vaccine signature sites, K169 in V2 and I307 in V3, associated with reduced infection risk in RV144, occurred less frequently in clade C panel viruses than in CRF01_AE viruses from Thailand. Increased resistance of pre-seroconversion viruses and evidence of antigenic drift highlights the value of using panels of very recently transmitted viruses and suggests that interventions may need to be modified over time to track the changing epidemic. Furthermore, high divergence such as that observed in the older clade C epidemic in southern Africa may impact vaccine efficacy, although the correlates of infection risk are yet to be defined in the clade C setting. Findings from this study of acute/early clade C viruses will aid vaccine development, and enable identification of new broad and potent antibodies to combat the HIV-1 C-clade epidemic in southern Africa.

## Author Summary

Vaccine and passive immunization prophylactic trials that rely on antibody-mediated protection are planned for HIV-1 clade C epidemic regions of southern Africa, which have amongst the highest HIV-1 incidences globally. This includes a phase 2b trial of passively administered monoclonal antibody, VRC01; as well as a phase 3 trial using the clade C modified version of the partially efficacious RV144 vaccine. The extraordinary diversity of HIV-1 poses a major obstacle to these interventions, and our study aimed to determine the implications of viral diversity on antibody recognition. Investigations using our panel of very early viruses augment current knowledge of vulnerable targets on transmitted viruses for vaccine design and passive immunization studies. Evidence of antigenic drift with viruses becoming more resistant over time suggests that these prevention modalities will need to be updated over time and that combinations of antibodies will be necessary to achieve coverage in passive immunization studies. We further show that it may be more difficult to obtain protection in the genetically diverse clade C epidemic compared to RV144 where the epidemic is less diverse, although it should be noted that the correlates of infection risk are yet to be defined in the clade C setting.

## Introduction

The development of effective biomedical intervention strategies to prevent HIV-1 infection remains a global priority. To support these efforts, two large immunization trials in high incidence, clade C epidemic regions in southern Africa are imminent. The first, a Phase 3 efficacy trial using a vaccine similar to the one used in the RV144 trial modified to include clade C antigens will be tested to determine if the protection observed in the RV144 vaccine trial in Thailand can be replicated in this high incidence setting (http://vaccineenterprise.org/content/P5Partnership). The second is a Phase 2b trial to evaluate if passive administration of the VRC01 monoclonal antibody, that targets the viral CD4 binding site (CD4bs), reduces HIV-1 acquisition [1]. Both interventions rely on the induction of HIV-specific antibodies against the HIV-1 envelope glycoprotein. HIV-1 is extraordinarily diverse, and evaluation of potential coverage by these intervention strategies would therefore need to take envelope diversity into account. As there is a severe HIV-1 transmission bottleneck that may affect viral phenotype [2–7], studies that aim to elucidate the target for active and passive immunization should ideally be done on viruses that are collected soon after transmission.

Although correlates of protection from HIV-1 infection are not fully understood, both neutralizing and non-neutralizing antibodies are known to play a crucial role. The importance of neutralizing antibodies is demonstrated in non-human primate models where passive administration of broadly neutralizing antibodies (bnAbs) conferred complete protection from simian-human immunodeficiency virus (SHIV) challenge [8,9]. While bnAbs with extraordinary coverage have been tested for safety in HIV-1 infected and uninfected humans [1], the VRC01 trial will be the first to evaluate the effectiveness of bnAbs for prevention. While a vaccine should ideally elicit bnAb responses, no such vaccine has been developed to date. However results from the RV144 efficacy vaccine trial in Thailand found that non-neutralizing antibodies may offer an alternative route to protection [10]. The HIV-1 envelope is comprised of several variable domains, and antibody responses directed to V1/V2 variable loops [10,11], as well as to V2 and V3 linear peptides [12], were inversely correlated with infection risk in RV144. Genetic signatures in breakthrough infections that were associated with reduced acquisition risk were also identified [11,13,14].

The HIV-1 global pandemic is highly diverse, comprising of many different clades (also known as genetic subtypes) and recombinant circulating forms (chimeras between more than one clade). Although neutralizing antibody responses elicited by one clade are generally effective at neutralizing viruses across clades, there is evidence of enhanced potency when clades are matched [15–19]. Even within a clade, diversity has been shown to have an effect illustrated by the fact that the clade-matched neutralization advantage was more pronounced in regions with lower viral diversity such as Thailand, compared to epidemics in southern Africa with higher viral diversity [15]. The effects of viral diversification on neutralization targets have also been seen by tracking the epidemic over time. Two cohorts in Europe, both predominantly infected with clade B, showed that HIV-1 has evolved at a population level to become more resistant to serum and bnAb neutralization over a twenty-year period [20–22]. This high diversity and increasing neutralization resistant phenotype is a potential problem for both active and passive immunization.

Immunization strategies need to block the virus(es) that establishes infection, and it is thus important to understand the properties of these transmitted founder viruses. There have been conflicting observations in cross-sectional studies as to whether viruses collected soon after transmission are more sensitive or more resistant to neutralization compared to viruses from chronic infection. In one study acute clade B viruses tended to be more sensitive to neutralization by VRC01, as well as pooled IgG from HIV-1 infected subjects compared to chronic viruses [5]. A separate clade C study generally detected no significant difference in neutralization susceptibility to VRC01 or sera [6]. In contrast to the clade B study, a large multi-clade study showed that early viruses were more neutralization resistant than late viruses when tested against clade-mismatched plasma, with no difference observed for clade-matched plasma [15]. Small sample size in some studies together with differences in study design and reagents all potentially confounded these studies, motivating for a large standard panel of viruses collected soon after transmission to more accurately evaluate targets of passive immunization studies and vaccines.

Here, using a large panel of 200 HIV-1 clade C Env-pseudotyped viruses generated from acute/early infection, we investigated the genotypic and neutralization properties of these viruses that may impact non-neutralizing and neutralizing antibody recognition. This collection is a good representation of viruses from southern Africa, the region of the world most affected by the HIV-1 epidemic and a major region for passive and active immunization efficacy trials. This panel, representing the largest collection of clade C Env-pseudotyped viruses from acute/early infection, is a valuable resource to the field, providing a reagent set that will enable establishing the cross-reactive potential of newly isolated monoclonal antibodies and the characterization of vaccine responses in the critically important HIV-1 clade C epidemic in southern Africa.

## Methods

### Ethics statement

The CAVIMC-CAVD HIV-1 Clade C Virus Neutralization Phenotype Study was reviewed and approved by the research ethics committee of the Faculty of Health Sciences of the University of Cape Town (168/2007; 513/2012). All participants provided written informed consent for study participation.

### Study participants

Samples used to generate functional *env* clones originated from Botswana (BW, n = 6), Zambia (ZM, n = 13), Malawi (MW, n = 23), Tanzania (TZ, n = 28), and South Africa (ZA, n = 130). South African samples originated from eight provinces: Western Cape (Cape Town, ZAwc n = 12), Eastern Cape (ZAec n = 2), North-West (ZAnw n = 7), Kwazulu-Natal (ZAkzn n = 68), Mpumalanga (ZAmp n = 6), Northern Cape (ZAnc n = 3), Limpopo (ZAlp n = 5) and Gauteng (Soweto and Johannesburg, ZAgp n = 27) (Table 1 and S1 Table). Of the 200, 199 were assumed to be sexually transmitted, and one was transmitted by breast feeding. Samples were catalogued as originating from an individual soon after transmission if they had a documented HIV-1 negative test within the previous 100 days, or if they were HIV-1 PCR positive and antibody negative at the time the sample was collected.

**Table 1:** Characteristics of the acute/early clade C panel virus donors by infection stage and country/region of origin.

			*Infection Stage*		
*Country & Provincial Origin*	*Abbreviation*	*Total*	*Pre-seroconversion* ^a^	*Indeterminate* ^b^	*Post-seroconversion* ^c^
Botswana	BW	6	1	5	0
Malawi	MW	23	13	7	3
Tanzania	TZ	28	2	1	25
South Africa	ZA				
Western Cape (Cape Town)	ZAwc	12	1	0	11
Eastern Cape	ZAec	2	0	2	0
North-West	ZAnw	7	1	0	6
Kwazulu-Natal	ZAkzn	68	19	8	41
Mpumalanga	ZAmp	6	5	1	0
Northern Cape	ZAnc	3	3	0	0
Limpopo	ZAlp	5	5	0	0
Gauteng (Johannesburg and Soweto)	ZAgp	27	14	4	9
Zambia	ZM	13	3	1	9
**Total**		**200**	**67**	**29**	**104**

^a^Pre-seroconversion/acute viruses (PCR positive but HIV-1 seronegative, Ab-) (Fiebig Stage I /II) (56).

^b^Indeterminate viruses (early seroconversion with an indeterminate western blot, Ab+/-) (Fiebig Stage III /IV).

^c^Post-seroconversion/early viruses (previous HIV negative diagnosis <100 days, Ab+) (Fiebig Stage V/VI).

### Single genome amplification (SGA) and sequencing

cDNA synthesis, followed by single genome amplification of *env* (~2.5kb), was performed according to a method described previously [4,23]. Both *env* strands were directly sequenced using an ABI PRISM 3100 Genetic Analyser using BigDye terminator reagents (Applied Biosystems, Warrington, UK), and sequence reads were assembled, edited and consensus sequences generated using Sequencher version 5.2.3 (Gene Codes Corporation, Ann Arbor, MI USA). Amplicons used for sequencing were generated using the limiting dilution approach, and for 90% (n = 179) of samples, at least five SGA derived sequences were generated per sample allowing for accurate transmitted founder consensus inference [24–26] (S1 Table).

### Pseudovirus production and infectivity assay

For pseudovirus production, full-length *rev-env* cassettes were cloned into one of two mammalian expression vectors, pcDNA 3.1 Directional/V5-His-TOPO (Invitrogen, Carlsbad, CA) or pTarget (Promega, Madison, WI). The amplicon sequence selected for cloning was the one closest to a participant’s consensus that was generated from at least five sequences. The resulting clones were sequenced to ensure an exact match to the original amplicon sequence and where cloned inserts differed from the parental sequence, mutagenesis was performed to ensure a match with the parental sequence. Env-pseudotyped viruses were generated by co-transfecting envelope clones with a clade B backbone pSG3ΔEnv (NIH AIDS Research and Reference Reagent Program) in HEK293T cells as previously described [27,28]. Pseudovirus functionality was determined by measuring luciferase expression after infecting TZM-bl cells (NIH AIDS Research and Reference Reagent Program, ARRRP). Relative luminescence units (RLUs) of ≥100,000 were considered ideal and 30,000 RLUs were accepted in cases where readings were 2.5 x the background; <2.5 times the background were considered negative.

### Co-receptor usage

Co-receptor usage was inferred by Geno2Pheno [29,30] and webPSSM [31,32] using a false positive rate (FPR) cut-off of 5 for Geno2pheno [33]. Viruses predicted to use CXCR4 were tested for co-receptor usage in the Trofile assay [34]. Viruses used in this entry assay were produced in an identical manner as described above except the HEK293T cells were co-transfected with *env* clones and the backbone, pNL4-3.lucRΔenv (NIH AIDS Research and Reference Reagent Program). Positive tropism controls RP1.12, an X4 virus [35], and QH0515.1, an R5 virus [36] were used. Viruses were normalized using the in-house p24 assay and standardized quantities of Env-pseudotyped viruses were tested for their ability to infect U87 cell lines expressing CD4 and either CCR5 (U87_R5) or CXCR4 (U87_X4) [37].

### Sequence analysis and phylogenetic inference

Sequences were aligned using GeneCutter (http://hiv.lanl.gov/content/sequence/GENE_CUTTER/cutter.html) [38] which enables the maintenance of alignments in codon space by employing HMMER [39] and is trained specifically for HIV-1 on a full-length genome alignment. Sequences were examined for evidence of inter-clade recombination using RIP [40], Rega HIV subtyping tool (400bp sliding window with 20bp steps size) [41] and with jpHMM [42]. Only sequences that were clade C throughout the entire *env* reading frame were included in the panel. For phylogenetic analysis and similarity comparisons, regions with more than 5% gaps were excluded. Candidate vaccine strains, Ce1086 (FJ444395 from Malawi in 2004); TV1 (AF391230 from South Africa in 1998); 96ZM651 (AF286224 from Zambia in 1996); and other clade C and clade C-related sequences from India and China (CRF07_BC, CFR08_BC and BC unique recombinants which were predominantly clade C across *env*, n = 59) were included as references. Phylogenies were computed using FastTree [43] on nucleotide sequences using the GTR substitution model. Gp120 amino acid distance calculations for comparing vaccine coverage and relatedness were determined using the HIVb substitution model in DIVEin [44]. The sequences from the placebo arm of the RV144 study (n = 66) were the same as those analyzed previously [13]. For investigation of the outward evolution of protein sequences, additional un-rooted phylogenies were computed in PhyML, version 3.0 [45] with the HIVb substitution model [46]. Outward evolution or divergence measured in branch length was calculated from protein phylogenies inferred in PhyML with trees rooted using the minimum sum of variance method [47]. Trees were visualized in FigTree v1.4.2 (http://tree.bio.ed.ac.uk/software/figtree/) and cluster support stated as >80% of a 100 resampled replicates. Env characteristics such as loop length, glycan density and net charge were determined for both conventional variable loops as well as for hypervariable regions within variable loops using a Los Alamos HIV-1 sequence database web tool which excises and characterizes either complete variable loops or hypervariable regions (http://www.hiv.lanl.gov/content/sequence/VAR_REG_CHAR). Weblogos of signature sites and linear peptide portions V2 (HXB2 161–179) and V3 (HXB2 300–322), were used to show amino acid frequency by position and were performed using a web tool at the Los Alamos HIV-1 database AnalyzeAlign (http://www.hiv.lanl.gov/content/sequence/ANALYZEALIGN/analyze_align.html). Signature analysis to distinguish between viruses from pre-seroconversion, indeterminate and post-seroconversion was performed using a phylogenetically corrected signature analysis strategy [48].

### Serum panel

Fifty-four serum samples were collected from antiretroviral (ARV) drug-naïve, chronically HIV-1-infected individuals originating from 3 SAAVI (South African AIDS Vaccine Initiative) clinical trial sites in South Africa: Durban, Kwazulu-Natal (n = 16, CAPRISA); Cape Town, Western Cape (n = 20; Desmond Tutu HIV Foundation, DTHF); and Soweto, Gauteng (n = 18, Perinatal HIV Research Unit, PHRU) (S2 Table). Serum samples were pre-screened for potency and breadth against three clade C pseudoviruses (CAP8.6F, CAP255.16 and Du156.12), a clade C consensus (ConC), a clade B pseudovirus (6535), a clade B consensus (ConB) and a single clade A pseudovirus (Q23.17). From this, a panel of 30 sera was selected, 10 per site, representing differing breadth (selecting some low, medium and high) for assaying against the 200 Acute/Early clade C panel (S3 Table). Serum samples were collected between March 2011 and October 2013 and all were heterologous, except for two samples from Durban. All analyses were adjusted to correct for the inclusion of these autologous measurements.

### Neutralization assays

Neutralization of Env-pseudotyped viruses was measured using the validated TZM-bl assay. Briefly, reductions in Tat-regulated firefly luciferase (Luc) reporter gene expression were measured in 96-well culture plates after a single round of virus infection in TZM-bl cells as described previously [27]. Assay stocks of Env-pseudotyped viruses were produced by transfection in HEK293T cells, as described above, and titrated in TZM-bl cells. Serum samples (inactivated at 56°C, for 1 h) and bnAbs were tested at 1:20 dilution and then at 3-fold dilutions up to seven times in duplicate wells for each dilution starting at either 10 μg/ml (PG9, PGT128) or 50 μg/ml (4E10) or 25 μg/ml (CAP256-VRC26.25). VRC01 was initially tested at10 ug/ml, and was later evaluated at 50 ug/ml. Neutralization was determined as the serum dilution or bnAb concentrations at which a 50% reduction in RLU was detected compared to virus control well RLUs, reported as the 50% inhibitory dilution (ID_50_) for serum samples and the 50% inhibitory concentrations (IC_50_) for bnAbs. In some cases we also report IC_80_ values for bnAbs (80% reduction in RLU). Background luminescence read from cells-only control wells was subtracted. Positive controls consisted of a HIV-1 IgG pool purified from five HIV-1 clade C-positive plasma samples (HIVIG-C) and a pool of purified IgGs from HIV-1 clade B positive plasma samples (HIVIG-B, NIH AIDS Research and Reference Reagent Program). bnAbs PG9 and PGT128 were provided by D. Burton (Scripps Research Institute). bnAbs VRC01 and CAP256-VRC26.25 were provided by J. Mascola (NIH Vaccine Research Center). bnAb 4E10 was commercially obtained from PolymunScientific (Klosterneuburg, Austria). All assays were conducted in laboratories adhering to Good Clinical Laboratory Practice (GCLP).

### Statistical analysis

Statistical comparisons were performed in GraphPad Prism 5.0 (GraphPad Prism version 5.00 for Windows, GraphPad Software, San Diego California USA, www.graphpad.com) or in RStudio, version 0.98.501 (R Core Team. 2013. R: a language and environment for statistical computing, http://www.r-project.org). Statistical significance was considered where p-values were ≤0.05. Differences in viral characteristics (neutralization susceptibility measured as ID_50_) and divergence of vaccine strains from the acute/early clade C and RV144 breakthrough viruses) were assessed using nonparametric Mann-Whitney tests for distributions between two un-paired groups. One-sided tests were performed where existing associations exist. Correlations of variable loop properties with IC_50_ values, phylogenetic branch length over time and ID_50_ values with branch length, were tested using Kendall’s rank correlation test as implemented in the R package Kendall v2.2, statistics provided for one-sided tests unless stated otherwise.

Differences in neutralization sensitivity between viruses over calendar time from three different periods (1998–2005, 2006–2007 and 2008–2010) were evaluated using a non-parametric Jonckheere-Terpstra test for trend among ordered groups (time interval groups in this case, with time periods pre-selected based on numbers). We tested an increase in neutralization resistance hypothesis.

Following the strategy used in Seaman et al., [16] tier categorization was performed by grouping k-means clustering whereby the 200 C-clade viruses were assigned to one of four subgroups (tier 1A, & B, tier 2 and tier 3) ranging from highly sensitive to resistant phenotypes. Subsequently, rank ordering of viruses according to their average log_10_ ID_50_ titers to further resolve tier 1B and tier 2 was performed [16,49]. We also tested the association between the ID_50_ titers and the early stage of infection, as well as the presence or absence of the glycan at position 332 using a Gaussian fixed-effect generalized model (GLM) with the titers as dependent variable and stage, the presence of the 332 glycan and year as independent variables.

### Nucleotide sequence accession numbers

Listed in S1 Table.

## Results

### Southern African HIV-1 acute/early clade C envelope panel

To generate a panel of 200 functional clade C envelope (*env* gene) clones from viruses collected soon after transmission, plasma samples were obtained from individuals estimated to be infected for less than a 100 days: 67 individuals were HIV-1 PCR positive but HIV-1 seronegative (pre-seroconversion, or Ab-) [50]; 29 were in early seroconversion with an indeterminate western blot (indeterminate, or Ab+/-); and the remaining 104 were HIV-1 seropositive with a negative diagnosis within the previous 100 days (post-seroconversion, or Ab+) (S1 Table). Samples originated from five southern African countries (South Africa, Botswana, Zambia, Tanzania and Malawi) (Table 1).

**Fig 1:**
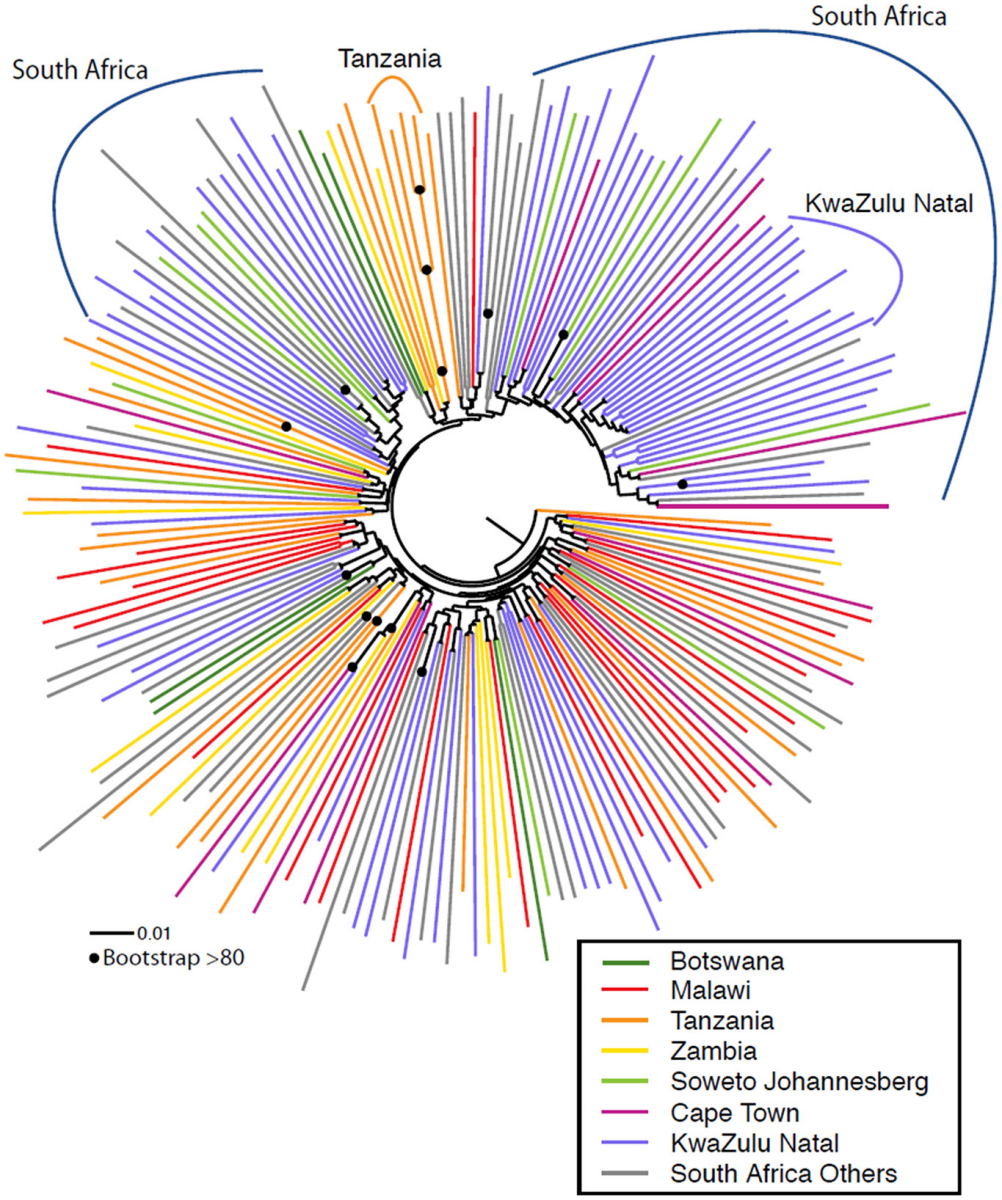
Maximum likelihood phylogenetic analysis of southern African clade C acute/early envelope nucleotide sequences (n = 200) (Table 1). Branches are colored according to country/region. Bootstrap values > 80% of 100 resampled replicates are illustrated as filled circles on nodes. South African samples from Soweto/Johannesburg (Gauteng province), Cape Town (Western Cape Province), and KwaZulu-Natal are highlighted in light green, red, and blue respectively, sequences from other locations in South Africa are shown in grey. Clades that were from the same geographic region are highlighted. Only one of these regional grouping (>2 sequence clusters) had strong bootstrap support (4 sequences from Tanzania, highlighted in orange).

Phylogenetic analysis showed that the viruses generally did not cluster according to country of origin, suggesting a generalized regional epidemic with intermixing of strains (Fig 1), although African and Asian C clade sequences form separate clades (S1 Fig). One bootstrap-supported cluster of four sequences, and two clusters of two sequences each, were identified from Tanzania; and seven clusters of two sequences each were identified from South Africa. The majority of South African sequences (68/130) originated from one particular region (KwaZulu Natal). We found no strong bootstrap-supported evidence of local founder effects within South Africa, although some clades consisting of sequences only of South African origin were evident, with one smaller clade of 10/68 sequences originated in Kwazulu-Natal observed (highlighted in Fig 1). S1 Fig provides a more detailed breakdown of all geographic origins within South Africa, and includes the vaccine strains that will be used in the upcoming trial, which are located in three phylogenetically distinct parts of the tree (S1 Fig).

Transmitted viruses almost exclusively utilize the co-receptor CCR5 [3,5,6]. In order to determine co-receptor phenotype in the clade C panel we inferred viral co-receptor usage based on the V3 loop amino acid sequence using two genotyping methods [29–33]. Of the 200 viruses, 192 were predicted to use CCR5 while eight were predicted as CXCR4-using by both methods (S4 Table). Experimental analysis using U87 cells expressing either the CXCR4 or CCR5 co-receptor [34], however found all eight to be exclusively CCR5-using with no evidence for dual tropism. Thus, all 200 clade C viruses were considered to be CCR5-tropic.

### Majority of clade C panel viruses have a moderately resistant tier 2 phenotype

**Fig 2:**
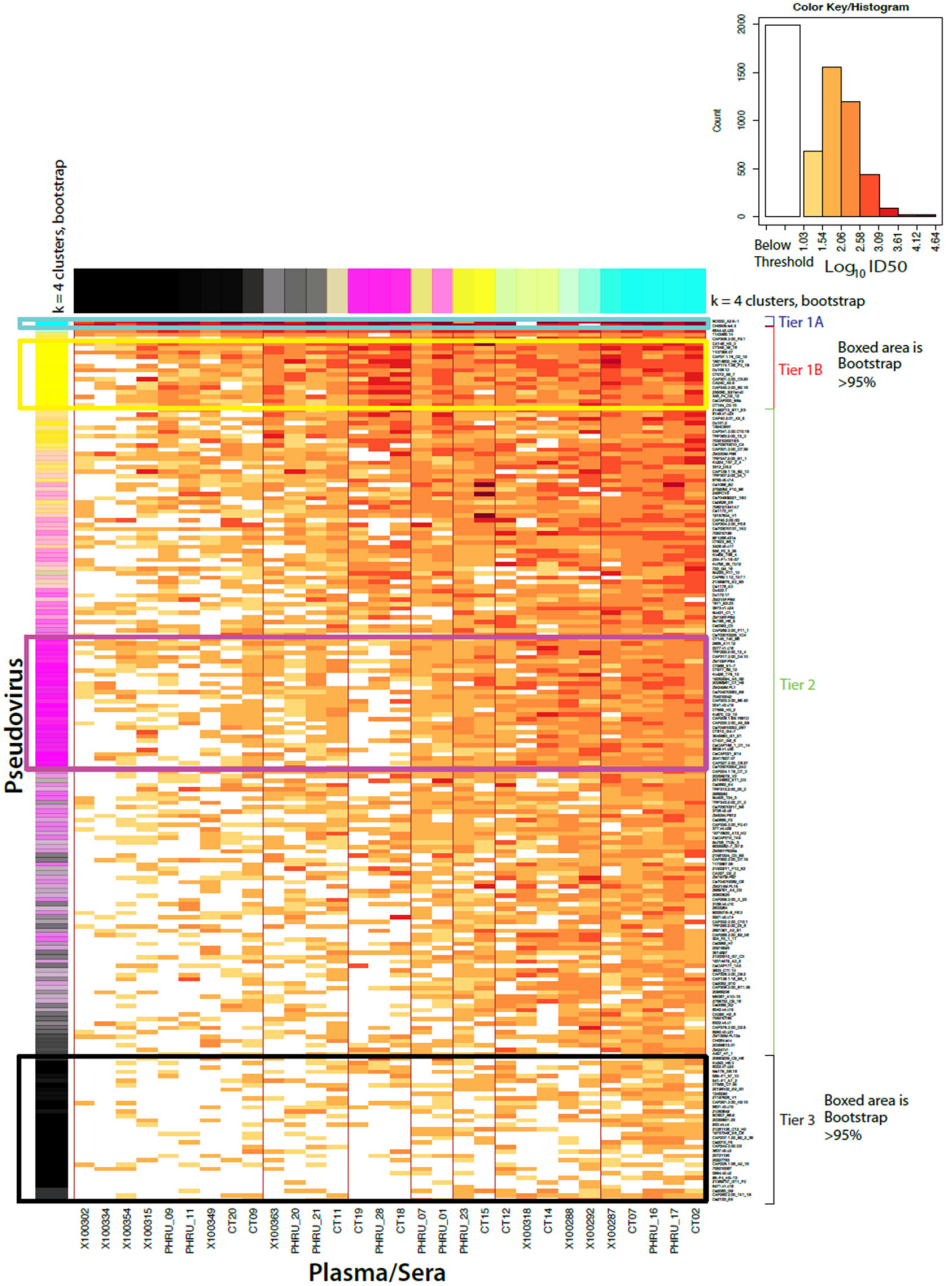
K-means (n = 4) neutralization clustering of clade C Env pseudoviruses for tier classification (n = 200). Neutralization sensitivity was assessed by assaying pseudoviruses against 30 sera from chronically infected individuals. Individual viruses are indicated to the right of the heatmap with individual serum listed at the bottom. Viruses are ranked according to respective sensitivities and categorized into tiers as determined by k-means clustering. Neutralization sensitivity (log_10_ ID_50_ titer) is denoted by color key provided in the inset. Viral isolates clustering, together with >95% probability, was performed to assess the stability of the Env pseudovirus clusters based on a random-with replacement resampling of the sera (rows), or to assess the stability of the serological clusters based on resampling of the viruses (columns), and were boxed on the heatmap indicating categorization into their respective tiers. Bootstrap support for clusters was determined by resampling the data sets from the individual serum and re-evaluating the k-means clusters 10,000 times giving an indication of how many times each virus was a member of the originally assigned tier classification in the resampled datasets. Env pseudoviruses that clustered with the assigned tier are shown in the column on the left as blue for tier 1A (very sensitive), yellow for tier 1B, magenta for tier 2 (intermediate), and black for tier 3 (very resistant). This follows the tier classification of Seaman et al (2010) [16]. Similarly sera potential is illustrated at the top with the most uniformly potent sera indicated as blue, potent sera as yellow, modestly potent as magenta, and least potent as black. Color intensity indicates the probability of falling within a cluster and the degree of blending the frequency of falling into each of the respective tiers, with strong unblended colors, associated with 95% bootstrap support, boxed. The poor resolution of the k- means bootstrapping emphasizes that the sensitivity of Envs is a continuum, and will be dependent on the specific sera or antibodies used for the evaluation.

**Fig 3:**
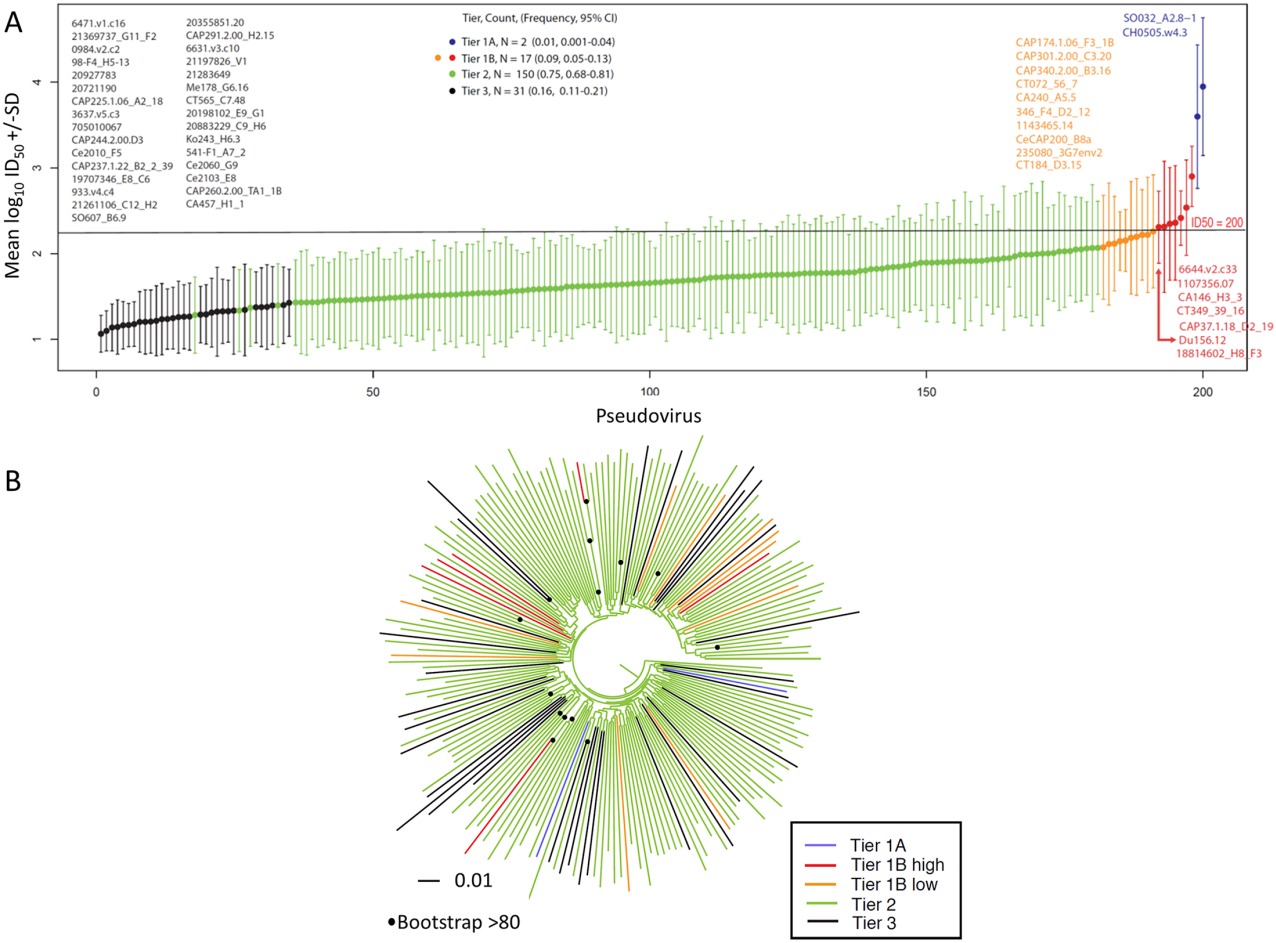
Geometric mean titer and tier 1A and 1B classification (n = 200). (A) Viruses are rank ordered according to neutralization sensitivity to 30 clade C chronic infection serum samples, from the least sensitive to the most sensitive along the x-axis by average log_10_ GMTs. Two viruses were classified as highly sensitive tier 1A and an additional 17 as above-average sensitive tier 1B. A previously determined cut off ID_50_ = 200, was used to distinguish between tier 1A and tier 1B is indicated on the graph, with tier 1B classified viruses above this cut off colored in red and those below in orange [16]. Twelve pseudoviruses classified by both Seaman et al., (2010) and this study were found to be discrepant (S1 Table): Du156.12 consistently falls near the boundary of the tier 2 and tier 1B, was classified as tier 1B here however was previously classified as tier 2; and ZM197M and SM109F were classified as tier 2 here, however were previously classified as 1B. (B) Maximum likelihood phylogenetic analysis of southern African clade C acute/early envelope nucleotide sequences (n = 200) with branches colored according to tier. Bootstrap values > 80% of 100 resampled replicates are illustrated as filled circles on nodes.

HIV-1 tier classification is a useful way to define a virus’ neutralization susceptibility profile, with tier 1 viruses being highly sensitive and tier 3 highly resistant to neutralization [16,49]. Neutralization assays were performed using 30 clade C HIV-1+ serum samples from chronic infection, which were prescreened to reflect sera with a range of neutralization potency and breadth (S2 and S3 Tables). Following a procedure published by Seaman et al (2010) [16], Envs were grouped using k-means clustering, and 4 clusters each representing a group of viruses with similar patterns of sensitivity were identified (Fig 2). This was used for an initial tier classification, with tier 1A, 1B and tier 3 forming highly significant and robust clusters, and tier 2 capturing everything between. This classification was recapitulated when neutralization sensitivity of each of the 200 viruses was measured as the geometric mean ID_50_ titer (GMT) determined for all sera against which each virus was tested (Fig 3A). The higher the GMT relative to other viruses in the panel, the more neutralization sensitive the virus. The rank of the titers closely reflected the tiers assigned by k-means clustering, although there was a small amount of intermixing between tier 2 and 3. The way Envs are grouped in a k means is generally highly dependent on the sera used for the evaluation, as is evident by the large number of Envs that sometimes are grouped with a more sensitive, sometimes with the more resistant cluster, depending on resampling. Consistent with previous approaches [16], the ambiguous calls that were not consistently highly sensitive in the bootstrap (tier 1), or consistently highly resistant (tier 3), were classified as tier 2. Fig 3A illustrates the continuum of geometric means.

The majority of viruses exhibited a moderately resistant tier 2 phenotype (75%, n = 150), whereas 8.5% (n = 17) were classified as possessing a more sensitive tier 1B phenotype, and 15.5% (n = 31) classified as possessing a more resistant tier 3 phenotype (Fig 3A). Phylogenetic analysis showed that the most sensitive or most resistant viruses generally did not cluster together (Fig 3B). To further interrogate if there were signatures in the viral sequences associated with tier phenotype, we considered every amino acid in each position using either a simple Fisher’s exact test testing for the association between the presence and absence of the amino acid and the phenotype of interest, and a phylogenetically corrected Fisher’s test (described fully in Gnanakaran et al, 2011) [51]. We identified no phylogenetic signatures associated with tier phenotype based on a false discovery rate (FDR) threshold of q < 0.2, however, using a simple Fisher’s test, a single association with tier 3 was identified, K683R. Lys (K) is the most common amino acid in this position. Arg (R) was enriched among Tier 3 viruses, found 16/30 times (53%), while among Tier 1 or 2 viruses it was present only 28/170 times (16%), (p-value of 0.00004, with a q = 0.04 for the full set of signature comparisons). This amino acid is located in the membrane-proximal external region (MPER) embedded in the 4E10 bnAb epitope, and the enrichment of R in Tier 3 viruses suggests that this position and region may be implicated in neutralization resistance.

The neutralization distribution of this clade C panel roughly approximated the tier distribution of a multi-clade panel of Env-pseudotyped viruses previously described (19%, 66% and 15% for tiers 1B, 2 and 3 respectively) [16]. Unique to our study was the identification of 1% (n = 2) of viruses with a highly sensitive tier 1A phenotype. This highly neutralization sensitive phenotype is not normally associated with circulating viruses. Both tier 1A envelope sequences matched a single genome derived amplicon, suggesting that these highly neutralization sensitive viruses do occur *in vivo*, although it remains possible that they were the result of an artificially introduced error during amplification, as both had a single mutation away from the derived transmitted founder sequence: the CH0505.w4.3 clone has a W680G mutation in the MPER [52] and the SO032_A2.8–1 clone had an E406G mutation in V4. We therefore do not know if these viruses were transmitted or if they evolved post-infection, or may have been the result of PCR error and we thus excluded them in all analysis pertaining to neutralization sensitivity.

### Neutralization sensitivity to polyclonal sera changed over the first 100 days of infection

There are conflicting data on whether viruses sampled in early infection are more or less sensitive to serum neutralization compared to viruses sampled later in infection. We investigated whether, over the first 100 days of infection, we could detect any difference in neutralization sensitivity. To select viruses that best reflected the virus *in vivo* that had recently traversed the transmission bottleneck, we included only envelopes generated using the single genome amplification (SGA) approach [23]; and excluded viruses that were classified as multi-variant transmission or for which there was insufficient information to classify their multiplicity of infection (S1 Table). This subset of 139 Env-pseudotyped viruses was classified into three groups according to time of infection where sequential gain of HIV-1 antibody responses was used as a marker of time from infection [50]: pre-seroconversion group (n = 58) (no detectable antibodies Ab-; estimated infection <15 days); indeterminate group (n = 26) (evolving antibody responses Ab+/-, estimated infection <37 days); and early post-seroconversion group (n = 55) (near/full seroconversion Ab+; estimated duration of infection < 100 days).

**Fig 4:**
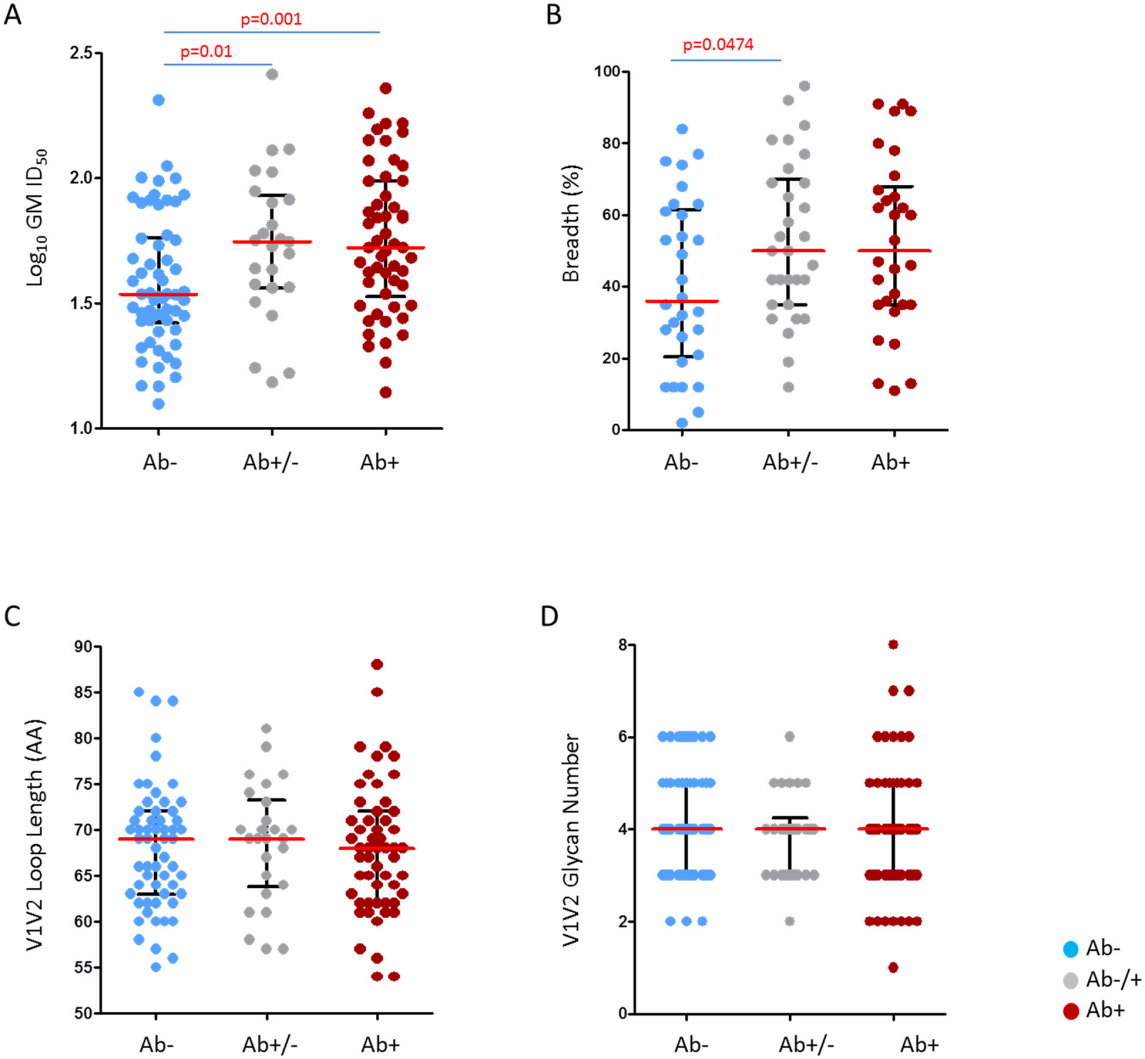
Comparison of neutralization susceptibility and viral characteristics of pre- and post-seroconversion clade C panel viruses. Pseudotyped clade C viruses were partitioned into three infection stage groups according to their sequential gain of HIV-1 specific antibody responses as a marker of time from infection; pre-seroconversion (Ab-) (n = 58) indicated in blue, indeterminate (Ab-/+) (n = 26) indicated in grey and post-seroconversion (Ab+) (n = 55) indicated in red. Each point represents the geometric mean of viruses tested for neutralization sensitivity using a panel of 30 South African sera. (A) Serum neutralization potency as measured by GMT, over the 30 sera/plasma included in this study; sera below the threshold of detection (dilutions of 1:20 was the limit tested) were given the value of 10. The two subgroups evident among the pre-seroconversion viruses did not cluster phylogenetically. One-sided Wilcoxon rank sum test employed with median values shown in red and interquartile ranges in black (B) Neutralization breadth per infection stage against 30 clade C serum samples measured as the percentage of viruses neutralized at ID_50_ > 1:20. Three very sensitive viruses, two tier 1A (SO032_A2.8–1, CH0505.w4.3) and one tier 1B, (6644.v2.c33), were excluded from the analysis. (C), (D) V1V2 loop amino acid length variation and glycan density per infection stage. Mann-Whitney two-sided tests used in panel B, C and D, with median values shown in red and interquartile ranges in black. Uncorrected p-values <0.05 are provided, however as four comparisons were made, only p-values <0.015 should be considered significant.

We found that pre-seroconversion viruses were more resistant to neutralization by the 30 clade C sera compared to post-seroconversion viruses, with pre-seroconversion viruses having a significantly lower GMT (p = 0.001, one-sided Wilcoxon rank sum test) (Fig 4A). There was no significant difference in GMT between the indeterminate group and the post-seroconversion group. To confirm that the result was real, given the possibility of error in measurements, we built an error model based on the many duplicated neutralization data points available [53]. We randomly resampled from the Gaussian distribution based on the observed error to add noise to our individual points, and generated 10,000 replicate datasets using the uncertainty model. For both comparisons, acute (Ab-) versus early (Ab+) or indeterminate (Ab +/-), more than 97% of replicates show similar trends as Fig 4A, i.e. the acute group is less sensitive than both indeterminate and early groups. Furthermore, using 10,000 replicates of artificial ID_50_ data generated as above, we also found that the acute group (Ab-) had higher normalized sum of ranks from the Wilcoxon rank sum test statistic than the indeterminate group (Ab+/-) in 99.8% replicates, and than the early group (Ab+) in 100% replicates (p < 10^−4^). This suggests that the results shown in Fig 4A are robust under realistic models of experimental uncertainty.

We also found that the pre-seroconversion viruses were neutralized less frequently by 30 clade C sera compared to the post seroconversion viruses (median breadth of 36% versus 50%; p = 0.0474; two sided Mann-Whitney test) (Fig 4B). This was largely driven by the resistant viruses as when only neutralization sensitive viruses were analyzed, there was no significant difference in sensitivity between pre-seroconversion, indeterminate and post-seroconversion viruses (median IC_50_ 85.90 μg/ml, 89.74 μg/ml and 95.06 μg/ml) (S8 Fig). This behavior was confirmed with purified IgGs from clade C infections (HIVIG-C), however no difference was observed when assayed against pooled purified IgGs collected from clade B infections (HIVIG-B) (S2 Fig), suggesting a possible clade-associated specificity may be driving this differential resistance pattern. Taken together, these data suggest that viruses present prior to seroconversion are less likely to be neutralized by clade-matched polyclonal HIV-1 sera than those viruses present post-seroconversion.

Several studies have shown that increases in length and glycosylation density across the V1, V2 and V4 regions of gp120 are associated with neutralization resistance [15,20,54–56]. We investigated whether these features could account for observed differences in neutralization sensitivity seen in the 200 clade C viruses. Similar to previous studies, when we analyzed all viruses together, we found that longer V1, V2 and V4 length, and higher glycan density were strongly associated with neutralization resistance (S3 Fig). However, we found no significant difference in V1, V2 and V4 loop length or glycan density between the pre-seroconversion, indeterminate and post-seroconversion groups of viruses (Fig 4C and 4D), indicating that observed differences in neutralization phenotype between these groups were not attributable to differences in variable loop length or glycan density.

### The glycan at position 332 and time post infection are independent predictors of neutralization resistance

**Fig 5:**
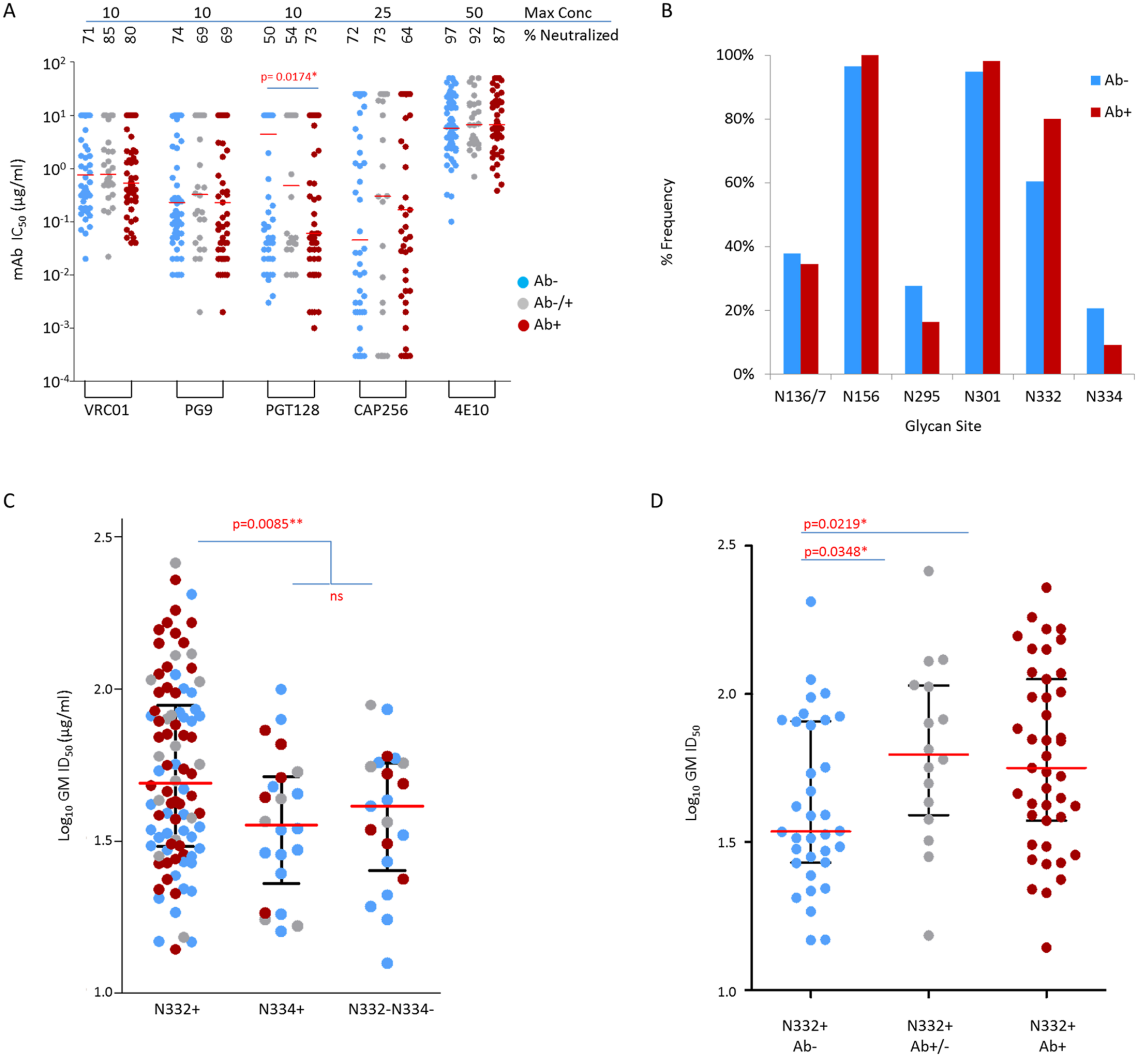
Role of the glycan at position 332 (N332+) in influencing neutralization susceptibility of clade C viruses. Each point represents the geometric mean of viruses tested for neutralization sensitivity using either bnAbs (A) or a panel of 30 South African sera (B and C). (A) Comparison of neutralization sensitivity of clade C viruses to bnAbs VRC01, PG9, PGT128, CAP256-VRC26.25 and 4E10. The highest concentration tested for each bnAb and the percentage of viruses neutralized (breadth) are indicated. Env pseudotyped clade C viruses (n = 139), from single infections for which SGA-derived sequences were available, were partitioned into three infection stage groups per sequential gain of HIV-1 specific antibody responses as a marker of time from infection; pre-seroconversion (Ab-), indeterminate (Ab-/+) and post-seroconversion (Ab+). Throughout, pre-seroconversion viruses are indicated in blue, with indeterminate in grey and post-seroconversion in red. Significant differences between medians indicated with the relevant p-value for a two-sided Mann-Whitney test. (B) Comparison of glycan frequency changes between pre-seroconversion (Ab-) and post-seroconversion (Ab+) infection stages for six sites comprising the glycan patch [57]. N-linked glycans were predicted according to the Nx(T/S) sequon, where x is not a proline. (C) Comparison of neutralization sensitivity between viruses categorized into those containing N332+ (n = 94), those with N334+ (n = 22) and those without a glycan at either position 332 or 334 (n = 21). Significance tested and shown with p-values provided for a Mann-Whitney, two-sided test. (D) A comparison of susceptibility using only viruses containing the N332 glycan, per infection stage. Dot plots show medians in red horizontal lines and interquartile ranges in black lines. Significance tested by non-parametric two-sided Mann-Whitney tests. Uncorrected p-values are provided.

We then investigated whether differences in neutralization phenotype could be attributable to differences in particular antibody specificities. Viruses were assayed with bnAbs targeting: the V2-glycan site (CAP256-VRC26.25 and PG9), the gp41 MPER epitope (4E10), the CD4 binding site (VRC01), and the V3/C3 glycan supersite (PGT128). Here IC_50_ was used, where low concentrations indicate increased viral sensitivity. For antibodies targeting the CD4bs, V2-glycan and MPER specificities we found no difference in neutralization susceptibility between pre-seroconversion and post-seroconversion viruses, suggesting that these epitope targets did not differ between the groups. However, pre-seroconversion viruses were significantly more resistant to neutralization by PGT128 compared to the post-seroconversion viruses (median IC_50_ of 4.42 μg/ml compared to 0.06 μg/ml respectively, p = 0.0174, Mann-Whitney two sided test) (Fig 5A), with 50% of pre-seroconversion and 27% of post-seroconversion viruses resistant to this bnAb.

PGT128 specificity is largely dependent on the presence of a glycan at position 332 [58,59], and similar to what we previously reported [59], there was an underrepresentation of this glycan in pre-seroconversion clade C viruses (Fig 5B). To determine the contribution of the 332 glycan in influencing neutralization resistance to polyclonal sera, we divided the 139 viruses into three groups: those with the glycan at position 332 (N332+), those with the glycan at position 334 (N334+) (the glycan at position 332 shifts to position 334 in some cases) and those with no glycan at either position 332 or 334 (N332-/N334-). Similar to previous studies, we found that viruses with the 332 glycan were indeed significantly more sensitive to serum neutralization compared to viruses lacking this glycan (median ID_50_ of 48.98 μg/ml, 35.73 μg/ml and 41.30 μg/ml for N332+, N334+ and N332-/N334- viruses respectively, p = 0.0085; Mann-Whitney two sided test) (Fig 5C). However, when we considered only viruses containing the 332 glycan, the pre-seroconversion viruses still had significantly lower sensitivity to serum neutralization (median ID_50_ of 34.36 μg/ml, 62.52 μg/ml, and 56.36 μg/ml, for Ab-, for Ab+/-, Ab+ respectively, p = 0.0219), indicating that the lack of the glycan at position 332 is not the sole determinant of increased resistance of pre-seroconversion viruses (Fig 5D). We then fitted a Gaussian fixed-effect generalized model (GLM) to test for an association between GMT with infection stage and the 332 glycan. Partitioning viruses into pre-seroconversion (Ab-) and indeterminate with post-seroconversion (Ab+/- and Ab+), the model showed that both the presence/absence of the 332 glycan and the stage of infection reliably predicted GMT, without any interaction between infection stage and glycosylation state (S4 Fig). The log_10_ GMT was on average 1.36 fold higher when the 332 glycan was present (p = 0.0094), and 0.72 lower when the sample was pre-seroconversion (p = 0.0034).

PGT128-like antibodies have been shown to recognize other glycans centered around the 332 glycan, the so-called high-mannose patch, which include glycans, N136/7, N156, N295, N301 and N334 [57]. N156 and N301 are conserved in clade C viruses and are therefore unlikely to contribute to differences in neutralization phenotype. N295 has been reported to substitute for N332 [60], but was found in higher frequencies in pre-seroconversion viruses and thus unlikely to be playing a role here. This together with N136/7, which occurs at similar frequencies in both pre-seroconversion and post-seroconversion groups, this could not account for increased neutralization resistance in the pre-seroconversion viruses (Fig 5B).

We sought to determine whether other genotypes were statistically associated with pre-seroconversion viruses. We identified a site in V5, G464, which was present in all post-seroconversion viruses in our panel, but in only 80% of pre-seroconversion/indeterminate viruses (p = 0.00024, q = 0.096; Fisher’s exact test). This site is located in the CD4 binding domain, however the relevance of this finding is not yet clear.

### Increased neutralization resistance as the clade C epidemic matures

**Fig 6:**
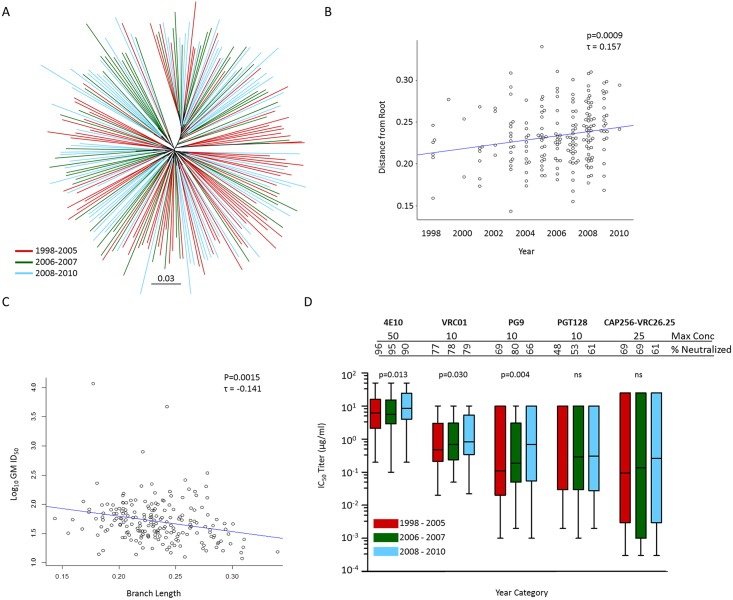
Diversification of the southern African clade C epidemic, over a period spanning 1998–2010, is associated with increased neutralization resistance. (A) Gp160 unrooted maximum likelihood phylogeny (PhymML) of protein sequences with sites greater than 5% gaps excluded, thereby disregarding hypervariable regions. Branches are colored according to sampling year category which was preselected based on numbers; warmer colors denoting earlier collected samples and cooler colors denoting samples collected later in the epidemic. (B) Protein ML phylogeny derived distances, estimated as branch lengths from root using the minimum sum of variance, correlated over time with calendar year. (C) Diversification (measured as branch length from root using the minimum sum of variance) inversely correlated with serum neutralization sensitivity. Rank correlations were performed using Kendall's rank correlation tau test. (D) Increasing resistance to antibody (bnAb) neutralization over the course of the clade C epidemic. bnAb susceptibility of clade C viruses from three distinct periods preselected based on numbers, 1998 to 2005 (n = 75), 2006 to 2007 (n = 55) and 2008 to 2010 (n = 70) tested against five bnAbs, VRC01, PG9, CAP256-VRC26.25, PGT128 and 4E10. The highest concentration tested for each bnAb and the percentage of viruses neutralized (breadth) are indicated. Placeholder constants of 50 μg/ml used for IC_50_ >50 μg/ml (4E10), 10 μg/ml used for IC_50_ >10 μg/ml (VRC01, PG9 and PGT128) and 25 μg/ml used for IC_50_ >25 μg/ml (CAP256-VRC26.25). Box plots show representative IC_50_ titer distributions for each bnAb tested across each period with median IC_50_ represented by horizontal lines. Differences of neutralization sensitivity between viruses over calendar time were evaluated using the Jonckheere-Terpstra test for trend. Assays using starting concentrations of 10μg/ml were used for VRC01 data reported here.

As this large collection of clade C viruses from soon after transmission was collected over 13 years, we sought to determine whether the southern African epidemic has increased in resistance to neutralization over time. For this we tested serum collected from clade C infected individuals between 2011 and 2013 against the clade C panel. We first investigated whether viruses diverged over this period, by determining Gp160 phylogenetic tree branch length (measured as distance from root) from 1998 to 2010. We found that early viruses (warmer colors) displayed shorter branches (closer to the root) compared to later viruses (cooler colors) (Fig 6A). Even when measuring divergence conservatively by determining protein distances on alignments excluding all hypervariable regions, we found a significant positive correlation of branch length over time (one sided Kendall’s τ = 0.157, p = 0.0009) (Fig 6B) indicating that the clade C epidemic diversified appreciably over the 13 year time period. We also observed a significant negative correlation of branch length with serum neutralization sensitivity, as measured by geometric mean ID_50_ titer (two sided Kendall’s τ = -0.141, p = 0.0015) (Fig 6C), but could find no direct correlation of polyclonal serum neutralization sensitivity over time (two sided Kendall’s τ = -0.017, p = 0.7338) (S5A Fig). We also found no significant association between variable loop length and glycan density over time (S5B and S5C Fig), suggesting that this trait has remained relatively constant. Taken together these data provides indirect evidence of diminishing sensitivity to within-clade sera over time.

We then evaluated changes in virus susceptibility to known bnAbs. Similar to polyclonal serum, we identified a significant negative association of branch length with bnAb neutralization sensitivity (measured in geometric mean IC_50_ titer for all bnAbs in aggregate) (Kendall’s τ = 0.1157, p = 0.0075) (S6A Fig). Viruses were grouped into three time periods according to date of collection to generate three groups of relevant sample size (1998–2005, 2006–2007 and 2008–2010). Although the first time period spanned 8 years, the majority of samples (>73%) were collected over a three-year period from 2003–2005. When we evaluated each of the five bnAbs individually, we found increased resistance as measured over three time periods to PG9, VRC01 and 4E10 (p = 0.013, p = 0.030 and p = 0.004 respectively, Jonckheere-Terpstra test), but not to PGT128 or CAP256-VRC26.25 (Fig 6D). Thus, we demonstrated that viruses are becoming more neutralization resistant to certain bnAbs as the epidemic progresses.

### VRC01 monoclonal antibody coverage in a clade C epidemic

**Fig 7:**
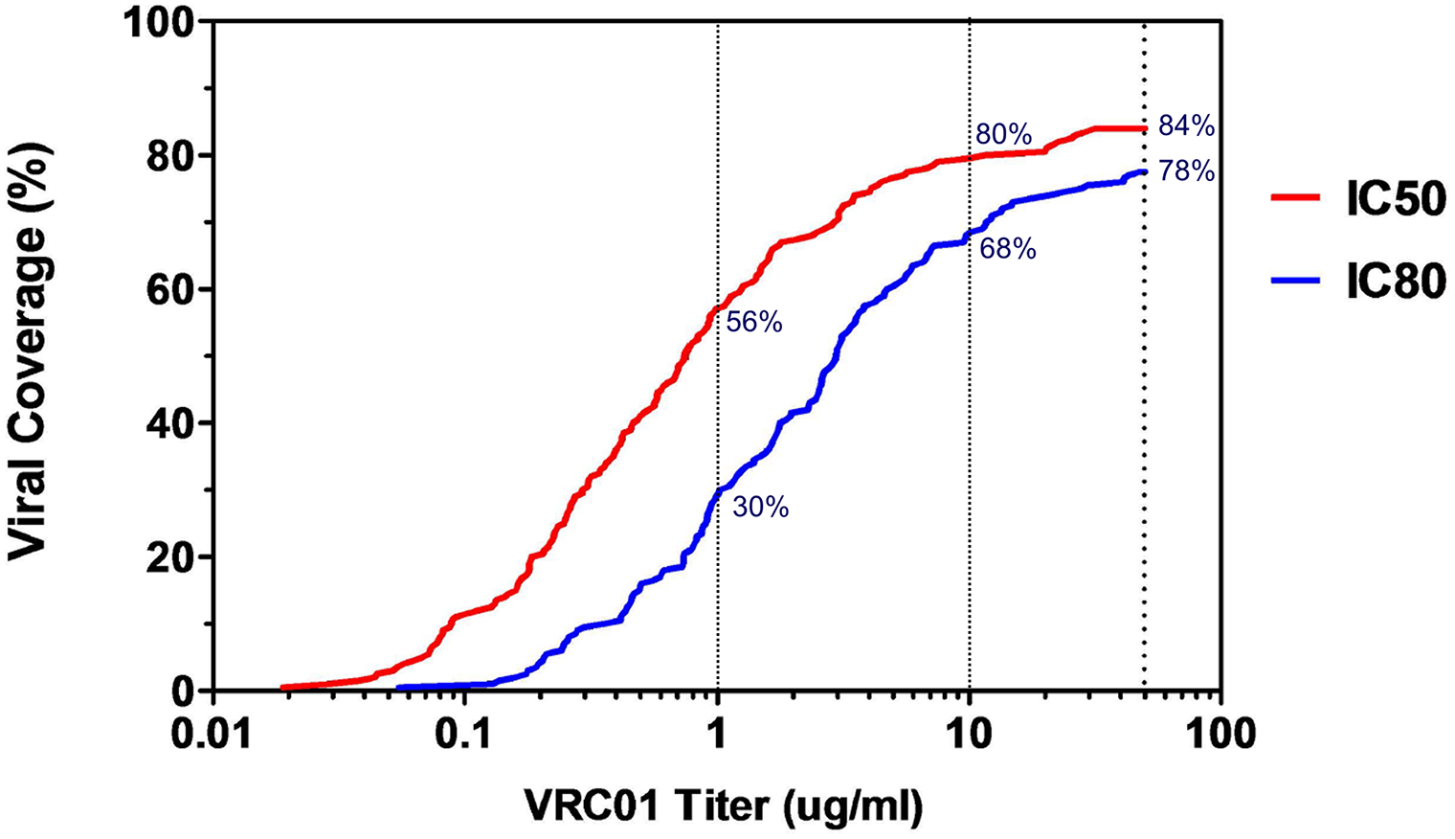
bnAb VRC01 coverage against clade C pseudoviruses viruses. Potency-breadth curves are presented for both IC_50_ in red and IC_80_ titers in blue. Dashed vertical lines indicate concentrations of 50 ug/ml, 10 ug/ml and 1 ug/ml tested. Assays using starting concentrations of 50μg/ml were used for VRC01 data reported here.

The role of VRC01 in preventing HIV acquisition is being evaluated in a Phase 2b study in Africa (ClinicalTrials.gov Identifier: NCT02568215), where trial participants will receive an intravenous infusion of VRC01 at a dose of 10 mg/kg or 30 mg/kg every 8 weeks. As VRC01 serum concentrations will decay over time between transfusions, we were interested in determining the effectiveness of this antibody at different concentrations including, 50 ug/ml, 10 ug/ml and 1 ug/ml. At these concentrations, 84%, 80% and 56% of the panel were neutralized respectively (IC_50_) (Fig 7). Assayed with the same concentration range, but measuring the concentration that resulted in 80% inhibition (IC_80_), VRC01 neutralized 78%, 68% and 30% of viruses, respectively. The median IC_50_ titer against sensitive viruses at <10 ug/ml was 0.42 μg/ml.

We have shown that viruses are becoming increasingly resistant to VRC01 as the epidemic matures, where overall potency at <10 ug/ml was shown to decrease from a median IC_50_ titer of 0.48 μg/ml to 0.69 μg/ml to 0.84 μg/ml over three time periods measured (1998–2005, 2006–2007 and 2008–2010) (Fig 6D). This was observed despite VRC01 breadth (percentage viruses neutralized at IC_50_ < 10 μg/ml) staying relatively constant over this time period (77%, 78% and 79% respectively).

### Protein divergence of clade C acute/early panel viruses from candidate vaccine strains

The RV144 trial in Thailand showed 31.2% vaccine efficacy with immune correlates of risk identified as antibodies predominantly against V1V2 region of gp120 [10]. Building on the success of RV144, a Phase 3 trial is planned for South Africa using vaccines similar to those used in RV144, however the canarypox vector has been modified to express the clade C (96ZM651) gp120 and the protein boost now comprises two clade C gp120 Env proteins (TV1 and Ce1086). To get insight into how such a vaccine will perform in the South African setting, we genotypically compared the clade C viruses to candidate C vaccines, and Thailand CRF01_AE breakthrough viruses to the AE gp120 immunogen used in the RV144 trial.

**Fig 8:**
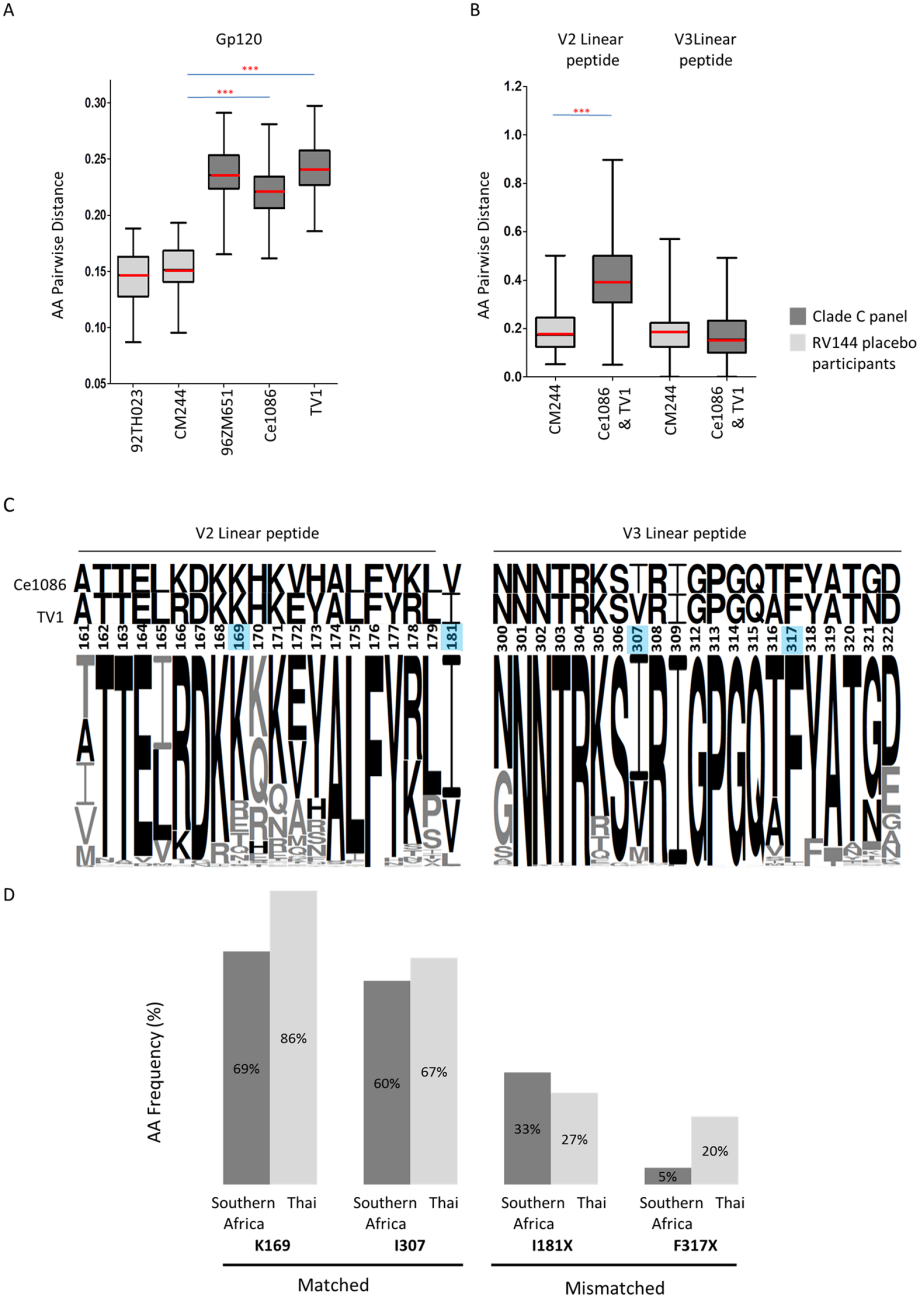
Relationship of clade C acute/early panel *env* genes/envelope proteins (n = 200) to clade C candidate vaccine strains compared to the relationship of CRF01_AE breakthrough viruses from RV144 (n = 66 placebo arm) to the RV144 vaccine. **(A)** Gp120 amino acid distances (excluding signal peptide) for clade C viruses to clade-matched vaccine prime- and boost-strains (96ZM651, TV1 and Ce1086); and CRF01_AE viruses from breakthrough infections in the placebo arm of RV144 to clade-matched RV144 vaccine strains (92TH023 and CM244). Box plots for RV144 distances in light grey and clade C distances in dark grey, with means in red. Significance shown in p-values provided for a two-sided Mann-Whitney test. **(B)** Amino acid distances across V2 (HXB2 161–179) and V3 (HXB2 300–322) linear B cell epitopes for clade C viruses as well as for RV144 placebo CRF01_AE viruses to respective clade-matched vaccine protein boost strains, Ce1086 and TV1 and CM244. Significance shown in p-values provided for a two-sided Mann-Whitney test. **(C)** Logo conservation plots across V2 and V3 linear B cell epitope peptides which includes vaccine signature sites as identified in RV144 [11,13]. These illustrate clade C acute/early panel sequence amino acid frequency and corresponding vaccine strain residue conservation. Residues shared between clade C and either vaccine strain Ce1086 or TV1 are shown in black with the remaining residues in grey. Positions of RV144 identified signatures associated with reduced infection risk K169, I181X, I307 and F317X are shaded in blue. **(D)** Comparison of RV144 signature site frequencies in acute/early clade C panel (southern Africa) compared to Thai CRF01_AE sequences from the placebo arm (Thai). In RV144, a match to the vaccine at position 169 (with a K) and at 307 (with I) was associated with protection; while a mismatch to the vaccine at positions 181 (I181X) and 317 (F317X) was associated with protection [11,13].

Phylogenetic comparison of the three clade C candidate vaccine strains showed that they fall within the 200 clade C panel sequences from southern Africa and cluster separately from clade C viruses sampled in India and China and recombinant CRF07_BC and CRF08_BC viruses from China (S1 Fig), suggesting they are broadly representative of the southern African epidemic. The mean gp120 amino acid sequence distance between the panel viruses and clade C candidate vaccine protein boost sequences ranged from 22.19% (95% CI of mean, 21.90%–22.48%) for Ce1086 to 24.15% (95% CI of mean, 23.85%–24.44%) for TV1. This was significantly greater distances than that observed in RV144, where the mean gp120 amino acid sequence distance between the CRF01_AE viruses from breakthrough infections in the placebo arm to the clade-matched vaccine immunogen, CM244 was 15.24% (95% CI 14.69%–15.79%) (Ce1086 and TV1 individually both to CM244 p<0.0001) (Fig 8A). Taken together the mean gp120 amino acid sequence distance of Ce1086 and TV1 to panel viruses was 23.14% (95% CI of mean 22.91%–23.37%), which was 8% more distant when compared to distances between CRF01_AE viruses and the RV144 vaccine immunogens.

### Divergence from the candidate vaccine V2 and V3 linear peptides

Binding antibodies to 19-mer V2 (HXB2 161–179) and 21-mer V3 (HXB2 300–322) linear peptides (so-called “hotspot” regions) were correlated with reduced risk of infection in RV144 [12]. We were interested in estimating the potential coverage of vaccines by determining the protein distance across V2 and V3 linear peptides between the C-clade vaccine boost proteins (TV1 and Ce1086) and the C-clade panel viruses.

We found high mean V2 amino acid distances of 40.51% (95% CI 39.13%–41.89%), with lower mean V3 amino acid distance of 17.10% (95% CI 16.20%–18.00%) (Fig 8B). Considering amino acids that were conserved in at least 90% of viruses across these hotspot regions, only 53% (10/19) of the V2 residues were conserved compared to 62% (13/21) in the V3 region (Fig 8C). We found that mean distances between the V2 hotspot region and viruses from the RV144 placebo group was significantly lower than in the clade C epidemic: 19.63% (95% CI 17.38%–21.88%) compared to 40.51% (95% CI 39.13%– 41.89%) respectively (p < 0.0001), but similar distance were observed in V3, 19.22% (95% CI 16.78%– 21.66%) compared to 17.10% (95% CI 16.20%–18.00%), Thailand and South Africa respectively) (Fig 8B). We cannot be sure however what the significance of this is, whether the absence of divergence in hotspots important in the RV144 trial will be critical for clade C. We have shown that the clade C epidemic is diverging over time and we were interested to see what effect this had on C-clade vaccines distances to clade C viruses across V2 and V3 hotspot regions. We found no significant difference between distances to 70 viruses collected after 2008 and the 130 remaining viruses collected during the preceding decade (S7 Fig), indicating that V2 and V3 hotspot regions are not diverging significantly over the period measured.

### Frequency of sites correlated with reduced infection risk in RV144 as found in clade C panel viruses

In the RV144 genetic sieve analysis, which compared sequences in the vaccine and placebo arm of breakthrough infections to the vaccine strains, a number of signature sites associated with reduced infection risk were identified [11,13,14]. Two of these genetic signatures (K169; I307) present in the RV144 vaccine strain were associated with increased vaccine efficacy against breakthrough viruses with matching amino acid residues at these positions, and have supportive experimental data to show that mutations found in vaccine breakthrough infections were associated with decreased antibody binding [11,13]. The protective signature K169 was present in both TV1 and Ce1086, whereas I307 was entirely absent. We determined the frequency of these protective signatures in our panel of clade C viruses, and compared them to the frequency in 66 viruses from the placebo arm of RV144 (Fig 8D). Both genetic signatures were found at a lower frequency in clade C viruses compared to RV144: the K169 site was found in 69% (95% CI 62%-75%) of clade C viruses compared to 86% (95% CI 78%–95%) of CRF01_AE viruses, and the I307 was found in 60% (95% CI 53%-67%) of clade C and 67% (95% CI 55%-78%) of CRF01_AE viruses respectively (Fig 8D). The variation in these sites in clade C is illustrated by a sequence logo (Fig 8C).

Two sites have been identified where a mismatch to the vaccine was associated with reduced risk of infection (I181X; F317X). The protective signature I181X was present in Ce1086 but absent in TV1, whereas neither Ce1086 nor TV1 contained the protective signature F317X. I181X was found at a similar frequency in clade C viruses compared to RV144 CRF01_AE viruses (33%, 95% CI 26%–40% compared to 27%, 95% CI 16%–38% respectively) whereas F317X was found at a lower frequency (5%, 95% CI 2%–8% compared to 20%, 95% CI 10%–30% respectively).

## Discussion

The extraordinary diversity of HIV-1 is a barrier to achieving protection in active and passive immunization studies. Several large prophylactic trials in clade C epidemic regions of Africa that rely on antibody-mediated protection are imminent, including passive immunization with the broadly neutralizing antibody (bnAb) VRC01, and a vaccine efficacy study of the RV144 regimen tailored for clade C. Using a large panel of 200 clade C pseudotyped viruses from acute/early infection, we observed that pre-seroconversion viruses were more resistant to antibody neutralization compared to post-seroconversion viruses. Additionally, we provide evidence of antigenic drift in certain bnAb targets including VRC01, which we estimated will only block ~80% of clade C viruses at its most efficacious dose. Furthermore, the higher divergence of clade C viruses from candidate clade C vaccines, compared to CRF01_AE viruses to the clade matched vaccine used in the RV144 vaccine, may make protection harder to achieve in clade C epidemic regions. Our study therefore provides a comprehensive analysis of viral traits that affect antibody recognition and interrogates how planned clinical trials may perform in the clade C epidemic of southern Africa.

Studies in non-human primate models have shown that vaccines have a limited window within which they can block infection, making the properties of pre-seroconversion viruses highly relevant [61]. The increased resistance of pre-seroconversion viruses seen in our study was only detected when viruses were assayed with clade C (and not clade B) polyclonal sera, suggesting clade-specific epitopes are either shielded or absent in these viruses. We propose that an under-representation of the N332 glycan (critical for the PGT128-like bnAbs), shown here and previously [59], is partially responsible for the more resistant phenotype. This is supported by the fact that antibody responses dependent on the N332 glycan are common in clade C sera [62]. The lack of the N332 glycan however was not the sole determinant of this resistant phenotype, as we still observed this effect even when only including viruses with the N332 glycan. Interestingly, an analysis using a large multi-clade panel of 219 viruses, tested against 170 polyclonal sera, also found viruses collected early in infection to be less sensitive to neutralization by polyclonal HIV-1 sera [15], suggesting that this phenomenon is more generalizable. However, the signal was only detected for inter-clade comparisons in Hraber et al., [15], which may have been due in part to a smaller sample size for within clade comparisons, and/or differences in the classification of viruses according to infection stage.

The reasons why pre-seroconversion viruses have a more resistant phenotype are unclear. It is possible that these traits provide the virus with a competitive advantage, either at the level of transmission, or in the early establishment of infection in the new host. Another possibility is that following transmission, and up to seroconversion replication in the absence of neutralizing antibodies, similar to cultivation in vitro [63], lead to adaptations that result in reduced shielding and thus the evolution of a more sensitive neutralization phenotype. Other studies examining viral genotypic properties shortly after transmission have also observed rapid adaptation with initial shortening and subsequent elongation of envelope variable loops as HIV-1 antibody responses evolve [64]. If this viral property is advantageous to establishing clinical infection, it may enhance transmission risk in a scenario where transmission predominantly occurs from donors who are in the acute/early stage of infection, which is thought to occur in roughly 50% of cases [65,66]. It will nevertheless be important to further elucidate the factors responsible for the increased resistance of pre-seroconversion viruses, to ensure that vaccines are designed to elicit antibodies that target the most vulnerable bnAb sites.

Persistent cycles of immune pressure have resulted in an accumulation of HIV-1 escape mutations at a population level. Recently, two studies, in predominantly clade B European cohorts, have shown that HIV-1 is also becoming increasingly neutralization resistant over time [20–22]. In our study we show for the first time that this is also occurring in the clade C heterosexual epidemic in southern Africa, where viruses are becoming increasingly resistant to some bnAbs, including those targeting the CD4bs (VRC01), V2-glycan (PG9-like but not CAP256-VRC26-like epitopes) and MPER (4E10). While Bunnick et al. [20] found that the viruses were increasing in loop length and glycan density as the epidemic progresses, similar to Bouvin-Pley et al, [21], we did not observe this genotypic change in our cohort. However, our study, as well as many others, have found these properties to be associated with neutralization resistance [2,15,55,67–69]. It is possible we did not detect significant changes in these properties due to our shorter sampling period of 13 years, as compared to a period of 20 years for other studies. These observations nevertheless, suggest that vaccine strains may need to be updated to be more representative of circulating diversity.

It was concerning to note that approximately 20% of clade C viruses in this panel were resistant to VRC01 and that they have become substantially less sensitive to this bnAb as the epidemic has matured, with an observed nearly doubling of concentration needed to reach 50% inhibition over thirteen years (Fig 6D). Thus, if antibodies are to be an effective prevention modality, they will need to have increased neutralization breadth, so as to reduce the impact of natural resistance. Furthermore, it may be necessary to use a mixture of antibodies that target multiple sites on the virus to both increase coverage and curb viral escape routes. This strategy is a focus of many research programs and there are several newer generation antibodies, and antibody combinations, currently in clinical development [9,70,71]. Utilizing this same virus panel with 15 bnAbs that target four regions (CD4 binding site, V1V2-glycan region, V3-glycan region and MPER), a comprehensive evaluation was performed to identify the best-in-class single antibodies, together with optimal combination of antibodies for HIV prevention and treatment [72].

The clade C version of the RV144 vaccine regimen is currently being tested for safety and immunogenicity, with Phase 3 trials due to start in South Africa in 2016. We analyzed envelope sequences at various sites or regions relevant to the correlates of risk in the RV144 vaccine trial, in order to consider how this vaccine may perform in the South African clade C epidemic relative to the vaccine used in the partially efficacious RV144 vaccine trial in Thailand. We found that the clade C gp120 protein boosts (Ce1086 and TV1) were generally representative of currently circulating viruses, however protein pairwise distance scores between the viruses and candidate vaccine boosts were approximately 8% greater than between RV144 vaccine immunogens and breakthrough viruses. Together with the low frequency of the RV144 signature sites, this may make vaccine protection harder to achieve in southern Africa compared to Thailand, a suggestion previously proposed by Hraber et al. based on neutralization properties [15]. However, in RV144 the major immune correlate of risk was V2 binding antibody responses, and while these responses were clade sensitive, they were found to be cross-reactive with clade C V2 peptides, suggesting that high diversity in V2 may have a limited impact [11,12]. Together these findings have important implications for future vaccine development although a major caveat associated with this conclusion is that the proposed correlates of risk identified in RV144 may be different in a clade C setting.

This clade C panel of acute/early Env pseudotyped viruses is the largest collection of functional clade C envelope clones available, and as such, provides a unique resource to the field. The samples are geographically representative of the southern African epidemic, and all samples were from acute/early infections. Together this makes them valuable reagents to support HIV-1 vaccine and passive immunization clinical trials, and important reagents for characterization of the breadth and potency of newly isolated bnAbs. However, one limitation of this work was that, due to the difficulty in obtaining very early samples, this study took many years to accumulate sufficient sample numbers, and only 63% of the panel was relatively current (obtained between 2006–2010).

In conclusion, elucidation of viral traits associated with resistance to antibody responses, and how these change over time, will be important to inform vaccine design efforts. This panel will be used to select representative viruses for evaluation of nAb responses in vaccine trials conducted in southern Africa. Furthermore, it has been used extensively to inform passive immunization studies and to prioritize bNAbs for clinical testing in clade C populations. This study has highlighted aspects that are highly relevant to vaccine design and provides insight into how efficacy vaccine trials, currently underway or imminent, will fare in this region of the world.

## Supporting Information

S1 Fig Maximum likelihood phylogenetic analysis of southern African clade C acute/early envelope nucleotide sequences (n = 200). Branches are colored according to country/regional origin. Fifty-nine clade C and clade C related sequences included in the tree from India and China are illustrated in grey. Circulating recombinant forms CRF07_BC, CFR08_BC (http://www.hiv.lanl.gov/content/sequence/HIV/CRFs/CRFs.html) as well as unique inter-clade B and C recombinant forms (URFs) that were clade C across Env are included. Southern African candidate vaccine strains, TV1, Ce1086 and 96ZM651 are indicated. Country abbreviations used are: Botswana (BW), Malawi (MW), Tanzania (TZ) Zambia (ZM) and South Africa (ZA). South African sequences were further partitioned by region and annotated by symbols: Eastern Cape (ZAec), Western Cape (ZAwc), Northern Cape (ZAnc), Mpumalanga (ZAmp), Kwazulu-Natal (ZAkzn), Limpopo (ZAlp), Gauteng (ZAgp) and North-West (ZAnw) (Table 1). Bootstrap values > 80% of 100 resampled replicates are illustrated as filled circles on nodes.
(TIF)

S2 Fig Variation in neutralization sensitivity of clade C viruses overall and per infection stage to IgG pools from clade B infected individuals (HIVIG-B) and from clade C infected individuals (HIVIG-C). Pseudotyped clade C viruses were partitioned into three infection-stage groups per sequential gain of HIV-1 specific antibody responses in the donors as a marker of time from infection; pre-seroconversion (Ab-) in blue, indeterminate (Ab-/+) in grey and post-seroconversion (Ab+) in red filled circles. Significant differences in sensitivity for Ab- and Ab+ viruses are shown as p-values for a two-sided Mann-Whitney test. Graphs show median IC_50_ values illustrated by horizontal lines in red. Higher IC_50_ titers reflect increased neutralization resistance.
(TIF)

S3 Fig Strong associations exist between clade C neutralization sensitivity and hypervariable loop properties such as loop length, glycan density and net charge. Associations tested between virus sensitivity and hypervariable loop properties; length (A); glycan density (B); and net charge (C). Significance of associations was tested using Kendall’s rank correlation tau. Three exceptionally sensitive viruses, two tier 1A (SO032_A2.8–1, CH0505.w4.3) and one tier 1B, (6644.v2.c33), were excluded from this analysis.
(TIF)

S4 Fig Modelling the contribution of N332 and infection stage on neutralization sensitivity of viruses. Viruses from pre-seroconversion are indicated in blue and those from post-seroconversion in red. Open and filled circles depict viruses without and with N332 respectively. Medians are shown by horizontal lines in red and interquartile ranges in black. Significant differences between medians (in red) indicated with the relevant p-value for a two-sided Mann-Whitney test.
(TIF)

S5 Fig Direct correlation of polyclonal sera neutralization sensitivity and Env characteristics over the course of the clade C epidemic. No discernible significant association of polyclonal sera neutralization (A) or Env characteristics, variable loop length (B) and glycan density (C) over time. Correlations were performed using Kendall's rank correlation tau test.
(TIF)

S6 Fig Diversification of the southern African clade C epidemic, over a period spanning 1998–2010 is associated with overall increased resistance to bnAb neutralization. (A) Protein ML phylogeny derived distances, estimated as branch lengths from root using the minimum sum of variance method, correlated to bnAb susceptibility measured as the geometric mean IC_50_ titer against all five bnAbs, VRC01, PG9, PGT128, CAP256 and 4E10 in aggregate. Placeholder constants of 50 were used for IC_50_ >50 μg/μl, >10 μg/μl >25 μg/μl. (B) Sensitivity to bnAb correlated to calendar year. Correlations tested with Kendall’s Tau.
(TIF)

S7 Fig Gp120 Amino Acid divergence of acute/early clade C viruses to vaccine inserts over the course of the clade C epidemic. No significant difference in Gp120 amino acid distances of panel viruses to vaccine inserts Ce1086 and TV1, when comparing viruses collected from earlier in the epidemic (before 2008, n = 130) to those collected later in the epidemic (after 2008, n = 70).
(TIF)

S8 Fig Comparison of neutralization susceptibility only for neutralization-sensitive clade C panel viruses. Viruses were partitioned into three infection stage groups according to their sequential gain of HIV-1 specific antibody responses as a marker of time from infection; pre-seroconversion (Ab-) (n = 58) indicated in blue, indeterminate (Ab-/+) (n = 26) indicated in grey and post-seroconversion (Ab+) (n = 55) indicated in red. Serum neutralization potency as measured by GMT. Three very sensitive viruses, two tier1A (SO032_A2.8–1, CH0505.w4.3) and one tier 1B, (6644.v2.c33), were excluded from the analysis. Mann-Whitney two-sided tests employed with median values shown in red and interquartile ranges in black.
(TIF)

S1 Table Description of 200 southern African acute/early clade C panel viruses. (DOCX)

S2 Table Pre-screening of 54 of chronic serum samples against a panel of seven pseudoviruses, including 4 clade C’s (CAP8.6F, CAP255.16, Du156.12 and a clade C consensus); 2 clade B’s (6535 and a clade B consensus); and 1 clade A (Q23.17). (DOCX)

S3 Table Demographic characteristics of the subset of thirty sera and plasma samples selected based on evidence for cross-reactivity, sufficient sample availability, and geographical representation. (DOCX)

S4 Table Co-receptor usage predictions and *in vitro* Trofile assay confirmation. (DOCX)
