# Correction: Features of Recently Transmitted HIV-1 Clade C Viruses that Impact Antibody Recognition: Implications for Active and Passive Immunization

**DOI:** 10.1371/journal.ppat.1006641

**Published:** 2017-09-25

**Authors:** Cecilia Rademeyer, Bette Korber, Michael S. Seaman, Elena E. Giorgi, Ruwayhida Thebus, Alexander Robles, Daniel J. Sheward, Kshitij Wagh, Jetta Garrity, Brittany R. Carey, Hongmei Gao, Kelli M. Greene, Haili Tang, Gama P. Bandawe, Jinny C. Marais, Thabo E. Diphoko, Peter Hraber, Nancy Tumba, Penny L. Moore, Glenda E. Gray, James Kublin, M. Juliana McElrath, Marion Vermeulen, Keren Middelkoop, Linda-Gail Bekker, Michael Hoelscher, Leonard Maboko, Joseph Makhema, Merlin L. Robb, Salim Abdool Karim, Quarraisha Abdool Karim, Jerome H. Kim, Beatrice H. Hahn, Feng Gao, Ronald Swanstrom, Lynn Morris, David C. Montefiori, Carolyn Williamson

There is an error in the caption for Fig 3, “Geometric mean titer and tier 1A and 1B classification (n = 200).,” panel A. Please see the complete, correct Fig 3 caption here.

**Fig 3. Geometric mean titer and tier 1A and 1B classification (n = 200)**.

(A) Viruses are rank ordered according to neutralization sensitivity to 30 clade C chronic infection serum samples, from the least sensitive to the most sensitive along the x-axis by average log10 GMTs. Two viruses were classified as highly sensitive tier 1A and an additional 17 as above-average sensitive tier 1B. A previously determined cut off ID50 = 200, was used to distinguish between tier 1A and tier 1B is indicated on the graph, with tier 1B classified viruses above this cut off colored in red and those below in orange [16]. Three of the twelve pseudoviruses classified by both Seaman et al., (2010) and this study were found to be discrepant (S1 Table): Du156.12 consistently falls near the boundary of the tier 2 and tier 1B, was classified as tier 1B here however was previously classified as tier 2; and ZM197M and SM109F were classified as tier 2 here, however were previously classified as 1B. (B) Maximum likelihood phylogenetic analysis of southern African clade C acute/early envelope nucleotide sequences (n = 200) with branches colored according to tier. Bootstrap values > 80% of 100 resampled replicates are illustrated as filled circles on nodes.
