# Supplementary figures and images for: Features of Recently Transmitted HIV-1 Clade C Viruses that Impact Antibody Recognition: Implications for Active and Passive Immunization
Source: PLoS Pathog. 2016 Jul 19;12(7):e1005742. doi: 10.1371/journal.ppat.1005742 (PMC4951126; doi:10.1371/journal.ppat.1005742)

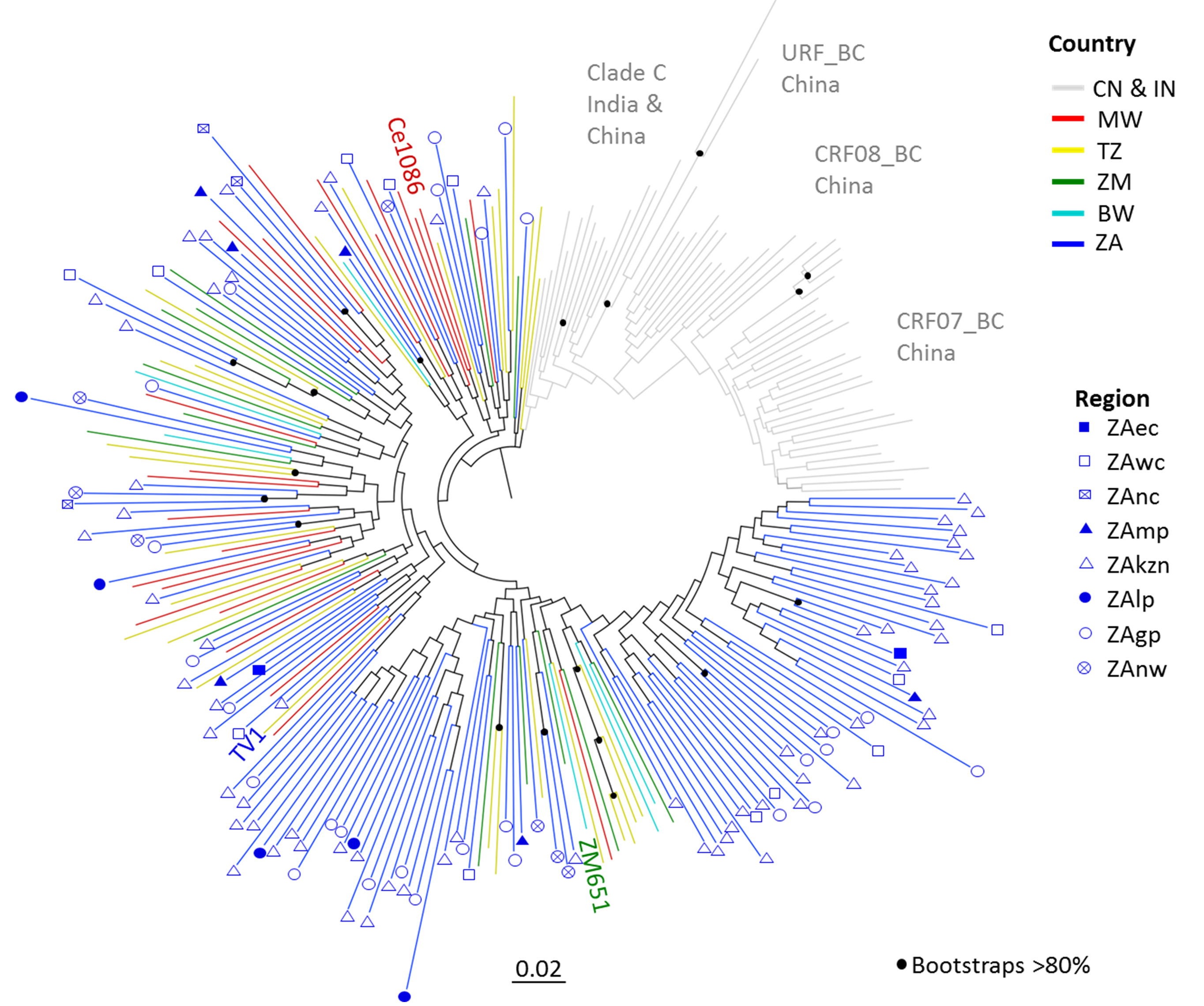

Supplement: S1 Fig — Branches are colored according to country/regional origin. Fifty-nine clade C and clade C related sequences included in the tree from India and China are illustrated in grey. Circulating recombinant forms CRF07_BC, CFR08_BC (http://www.hiv.lanl.gov/content/sequence/HIV/CRFs/CRFs.html) as well as unique inter-clade B and C recombinant forms (URFs) that were clade C across Env are included. Southern African candidate vaccine strains, TV1, Ce1086 and 96ZM651 are indicated. Country abbreviations used are: Botswana (BW), Malawi (MW), Tanzania (TZ) Zambia (ZM) and South Africa (ZA). South African sequences were further partitioned by region and annotated by symbols: Eastern Cape (ZAec), Western Cape (ZAwc), Northern Cape (ZAnc), Mpumalanga (ZAmp), Kwazulu-Natal (ZAkzn), Limpopo (ZAlp), Gauteng (ZAgp) and North-West (ZAnw) (Table 1). Bootstrap values > 80% of 100 resampled replicates are illustrated as filled circles on nodes. (TIF) [file ppat.1005742.s001.tif]

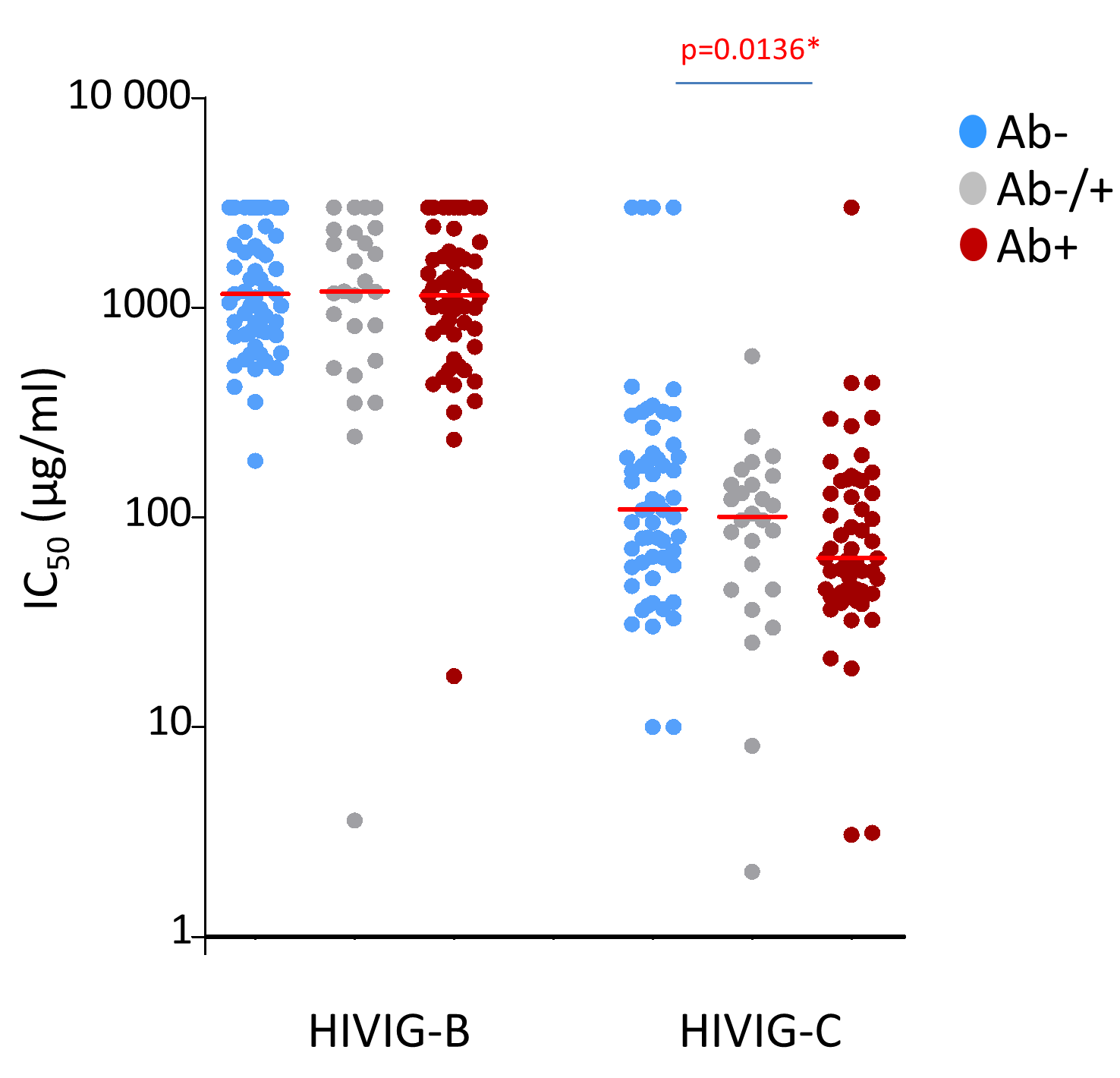

Supplement: S2 Fig — Pseudotyped clade C viruses were partitioned into three infection-stage groups per sequential gain of HIV-1 specific antibody responses in the donors as a marker of time from infection; pre-seroconversion (Ab-) in blue, indeterminate (Ab-/+) in grey and post-seroconversion (Ab+) in red filled circles. Significant differences in sensitivity for Ab- and Ab+ viruses are shown as p-values for a two-sided Mann-Whitney test. Graphs show median IC50 values illustrated by horizontal lines in red. Higher IC50 titers reflect increased neutralization resistance. (TIF) [file ppat.1005742.s002.tif]

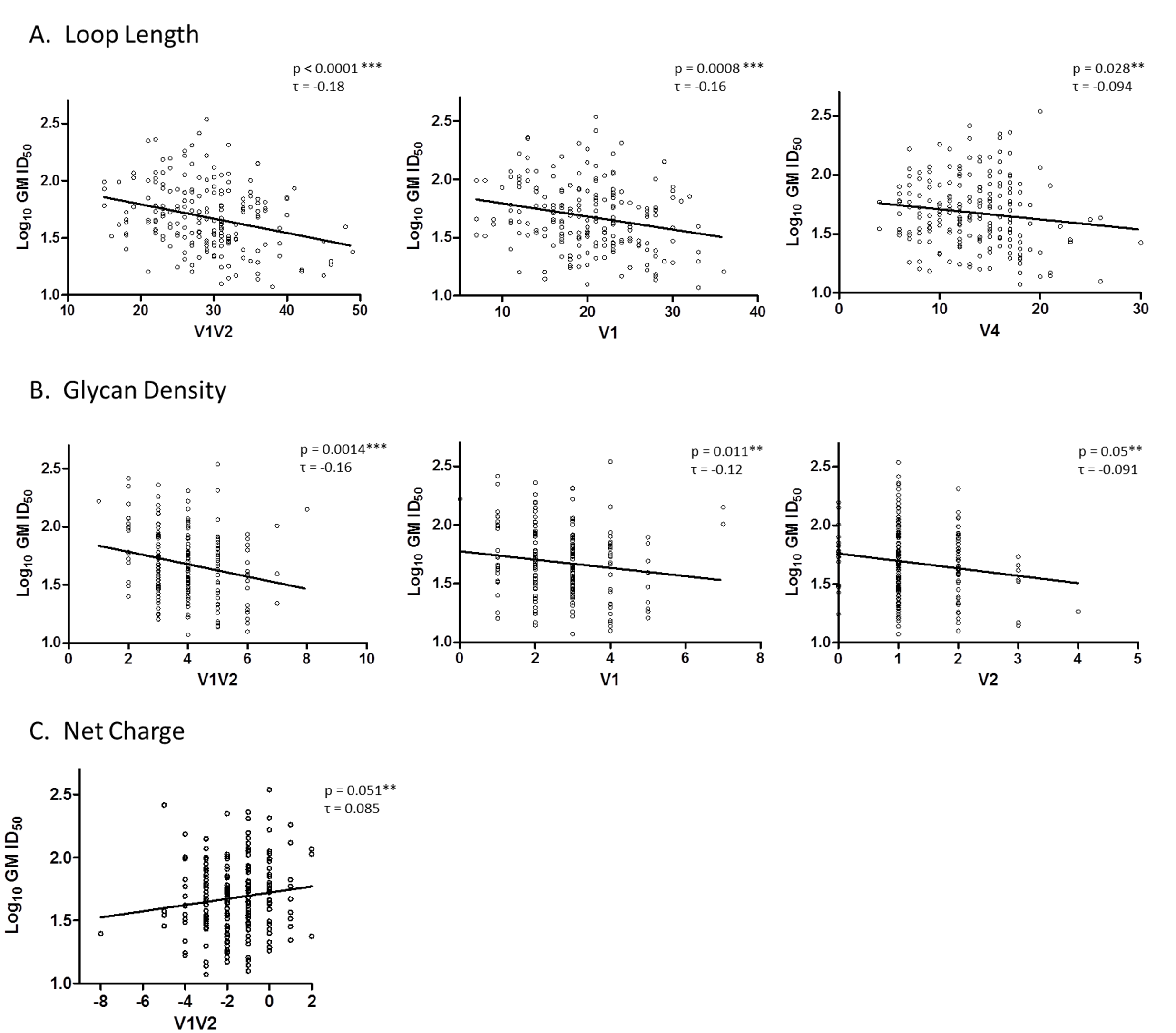

Supplement: S3 Fig — Associations tested between virus sensitivity and hypervariable loop properties; length (A); glycan density (B); and net charge (C). Significance of associations was tested using Kendall’s rank correlation tau. Three exceptionally sensitive viruses, two tier 1A (SO032_A2.8–1, CH0505.w4.3) and one tier 1B, (6644.v2.c33), were excluded from this analysis. (TIF) [file ppat.1005742.s003.tif]

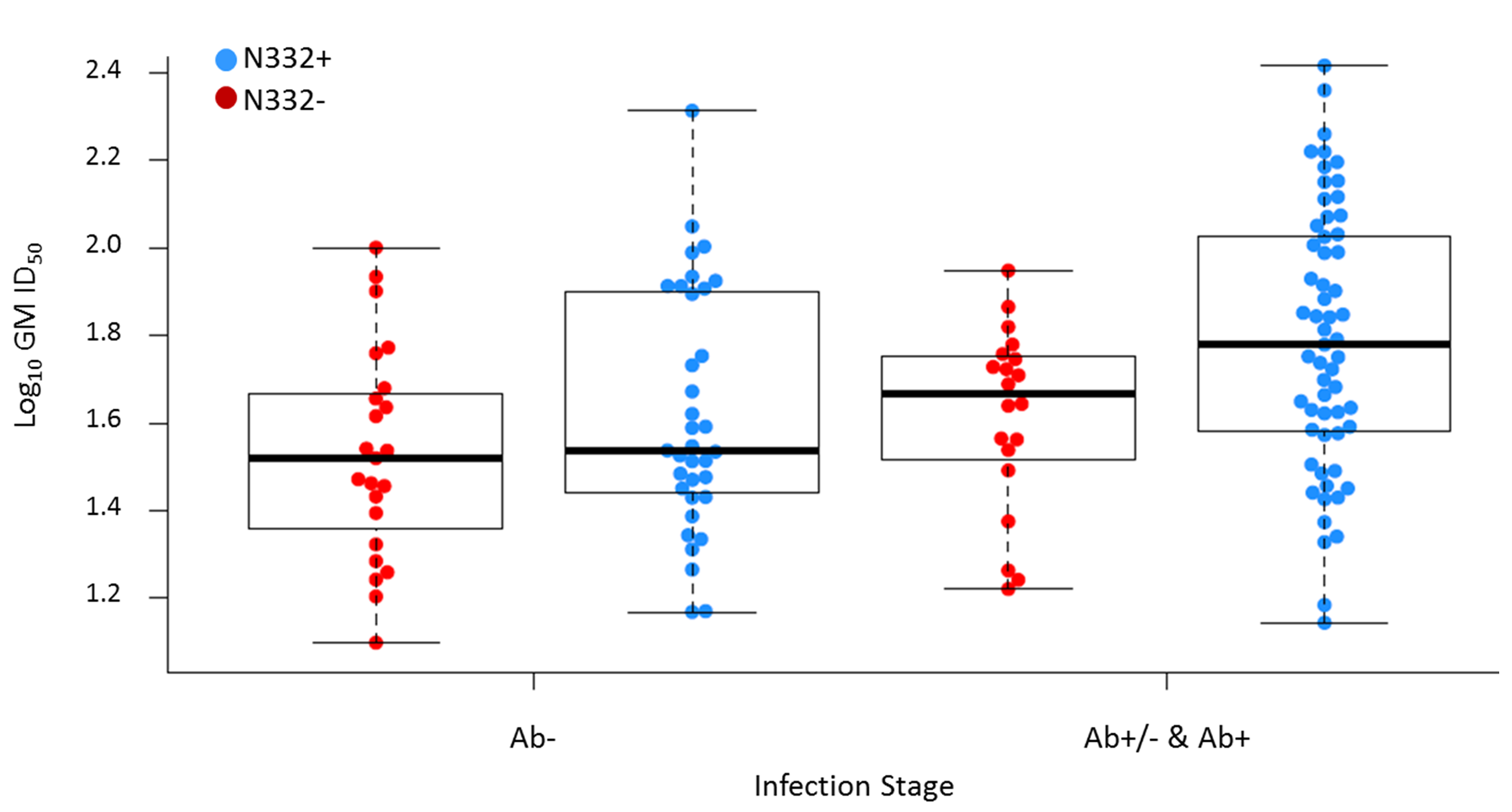

Supplement: S4 Fig — Viruses from pre-seroconversion are indicated in blue and those from post-seroconversion in red. Open and filled circles depict viruses without and with N332 respectively. Medians are shown by horizontal lines in red and interquartile ranges in black. Significant differences between medians (in red) indicated with the relevant p-value for a two-sided Mann-Whitney test. (TIF) [file ppat.1005742.s004.tif]

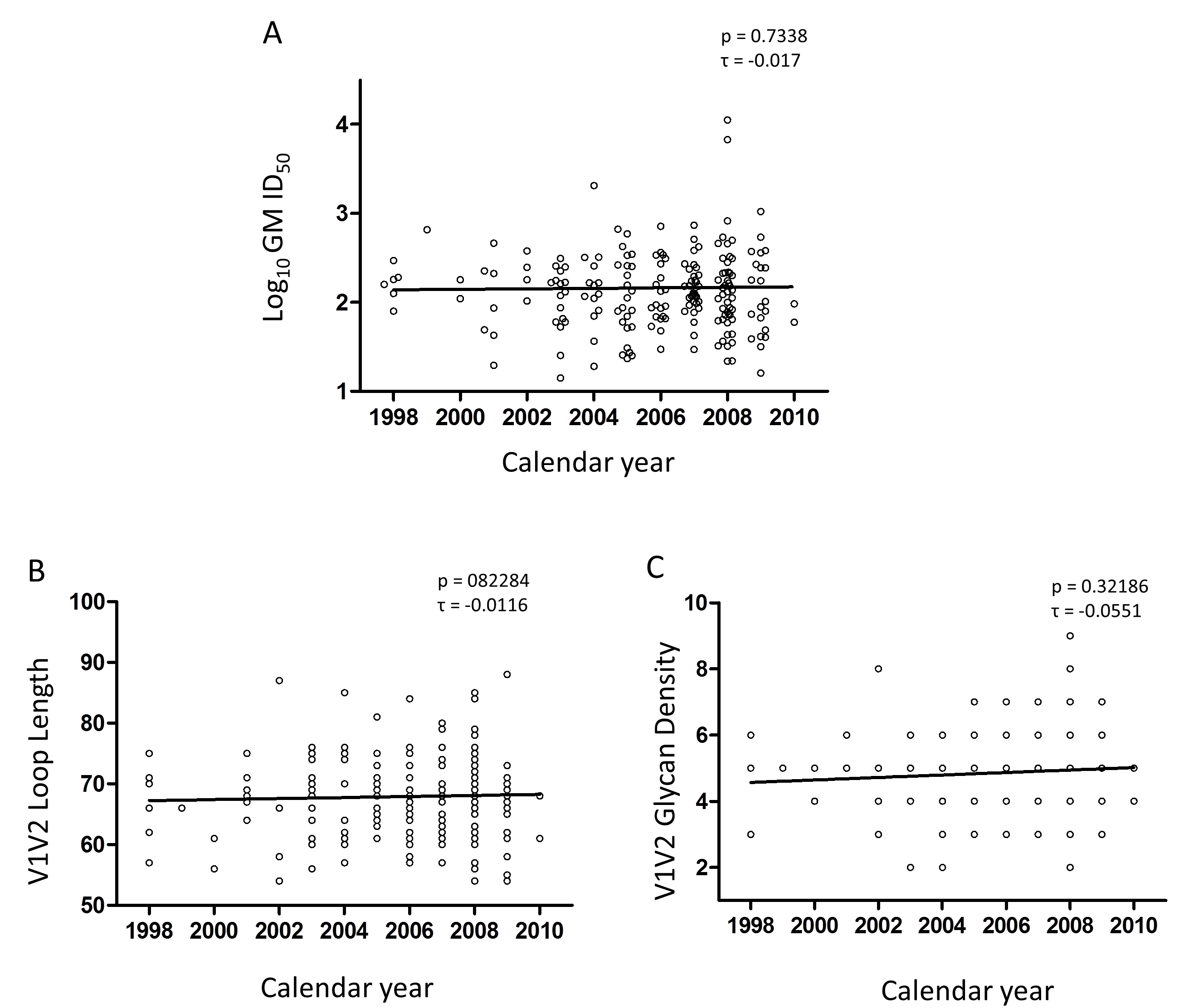

Supplement: S5 Fig — No discernible significant association of polyclonal sera neutralization (A) or Env characteristics, variable loop length (B) and glycan density (C) over time. Correlations were performed using Kendall's rank correlation tau test. (TIF) [file ppat.1005742.s005.tif]

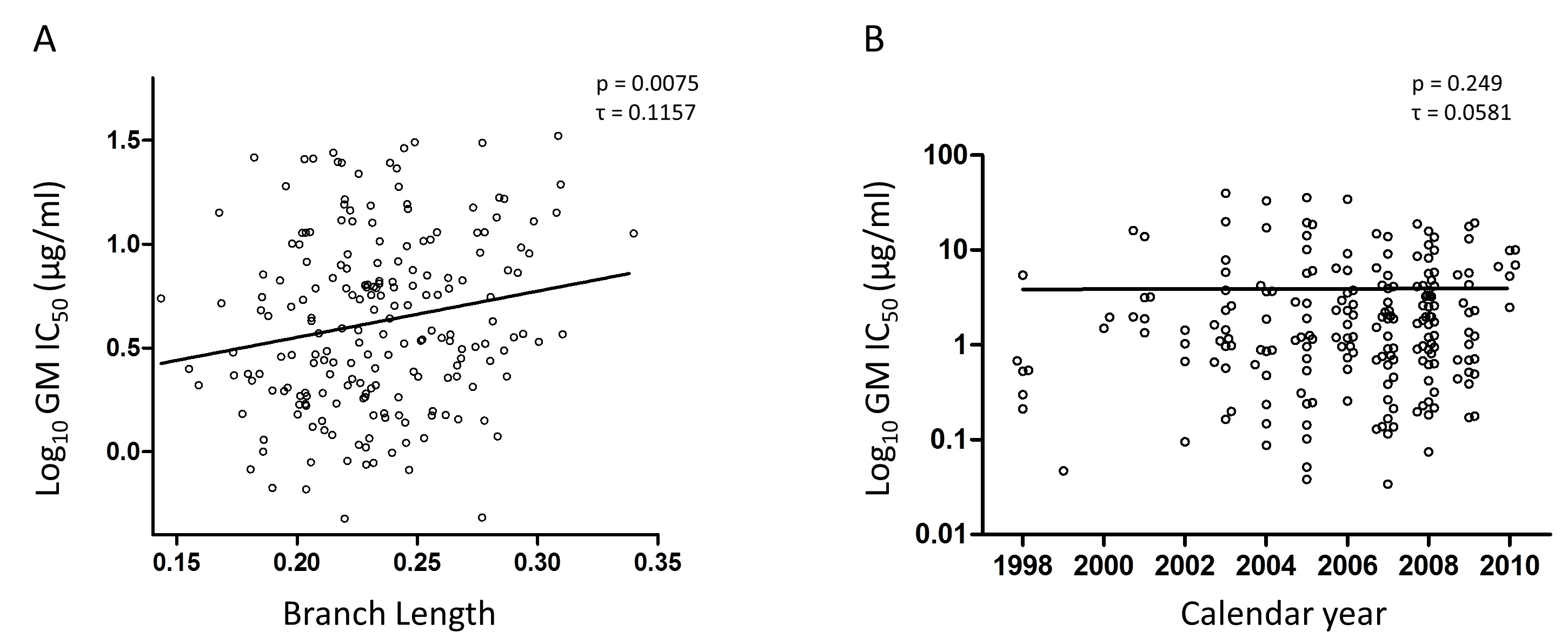

Supplement: S6 Fig — (A) Protein ML phylogeny derived distances, estimated as branch lengths from root using the minimum sum of variance method, correlated to bnAb susceptibility measured as the geometric mean IC50 titer against all five bnAbs, VRC01, PG9, PGT128, CAP256 and 4E10 in aggregate. Placeholder constants of 50 were used for IC50 >50 μg/μl, >10 μg/μl >25 μg/μl. (B) Sensitivity to bnAb correlated to calendar year. Correlations tested with Kendall’s Tau. (TIF) [file ppat.1005742.s006.tif]

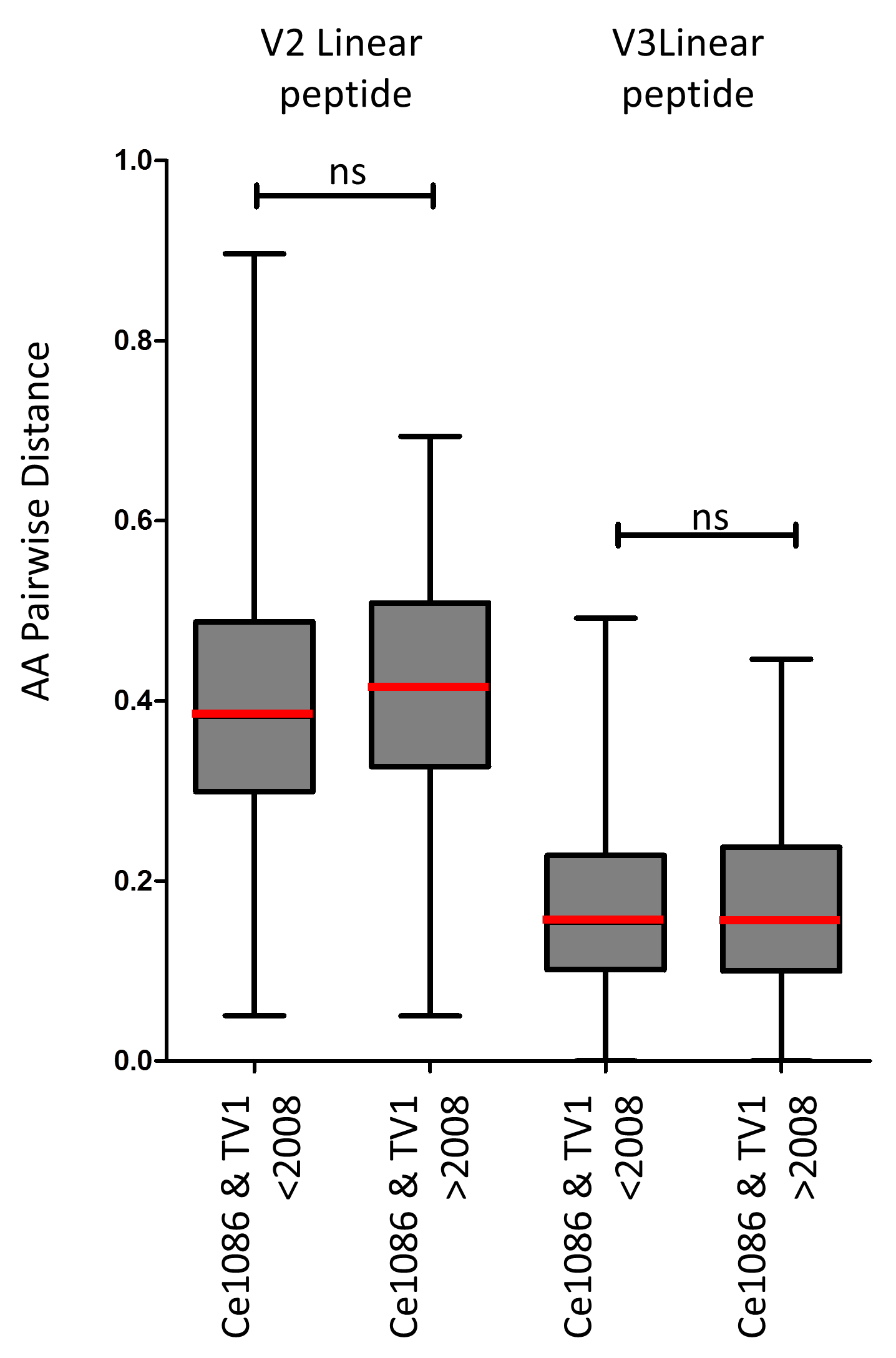

Supplement: S7 Fig — No significant difference in Gp120 amino acid distances of panel viruses to vaccine inserts Ce1086 and TV1, when comparing viruses collected from earlier in the epidemic (before 2008, n = 130) to those collected later in the epidemic (after 2008, n = 70). (TIF) [file ppat.1005742.s007.tif]

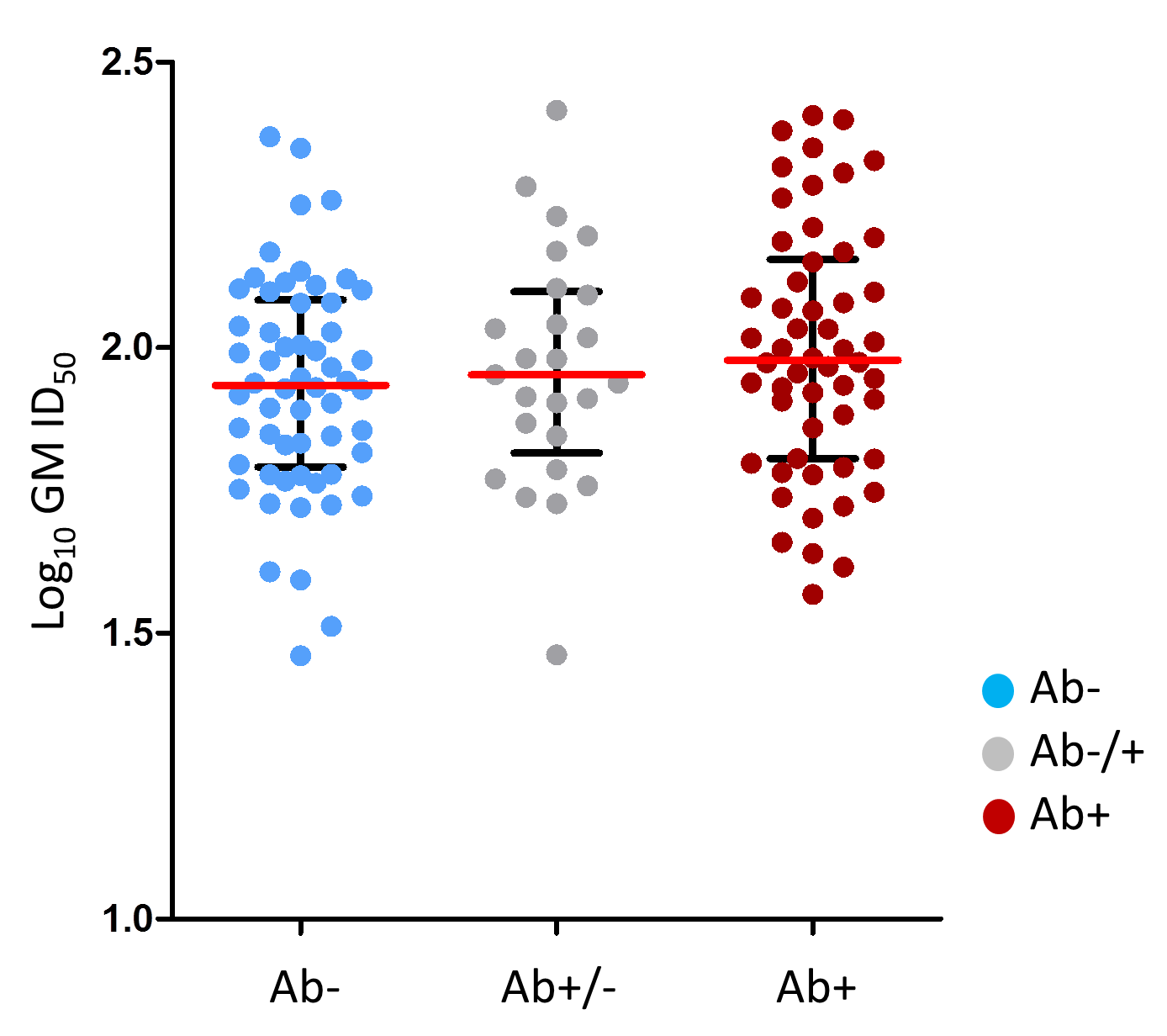

Supplement: S8 Fig — Viruses were partitioned into three infection stage groups according to their sequential gain of HIV-1 specific antibody responses as a marker of time from infection; pre-seroconversion (Ab-) (n = 58) indicated in blue, indeterminate (Ab-/+) (n = 26) indicated in grey and post-seroconversion (Ab+) (n = 55) indicated in red. Serum neutralization potency as measured by GMT. Three very sensitive viruses, two tier1A (SO032_A2.8–1, CH0505.w4.3) and one tier 1B, (6644.v2.c33), were excluded from the analysis. Mann-Whitney two-sided tests employed with median values shown in red and interquartile ranges in black. (TIF) [file ppat.1005742.s008.tif]
