# Supplementary material for: Features of Recently Transmitted HIV-1 Clade C Viruses that Impact Antibody Recognition: Implications for Active and Passive Immunization
Source: PLoS Pathog. 2016 Jul 19;12(7):e1005742. doi: 10.1371/journal.ppat.1005742 (PMC4951126; doi:10.1371/journal.ppat.1005742)
Supplement: S1 Table — (DOCX) [file ppat.1005742.s009.docx]

**TABLE S1** Description of 200 Southern African Acute/Early Clade C Panel viruses

| *Accession Number* | *Env Clone* | *Tier* | *Country & Provincial Origin^a^* | *Fiebig Stage or minimal time from infection* | *Infection Stage Classification^c^* | *Infection year* | *Nucleic Acid Amplification Method^d^* | *Multiplicitiy of infection^e^* | *Donating Institute or Network^f^* |
| --- | --- | --- | --- | --- | --- | --- | --- | --- | --- |
| AY423971 | ZM55F.PB28a | 2 | ZM | ≤VI | E | 1998 | LDA | NA | Zambia-Emory HIV Research Project |
| AY423984 | ZM53M.PB12* | 2 | ZM | ≤VI | E | 2000 | LDA | NA | Zambia-Emory HIV Research Project |
| AY424079 | ZM135M.PL10a | 2 | ZM | ≤VI | E | 1998 | LDA | NA | Zambia-Emory HIV Research Project |
| AY424138 | ZM109F.PB4* | 2 | ZM | ≤VI | E | 2000 | LDA | NA | Zambia-Emory HIV Research Project |
| AY424163 | ZM106F.PB9 | 2 | ZM | ≤VI | E | 1998 | LDA | NA | Zambia-Emory HIV Research Project |
| DQ388514 | ZM249M.PL1* | 2 | ZM | II | A | 2003 | LDA | S | Zambia-Emory HIV Research Project |
| DQ388515 | ZM197M.PB7* | 2 | ZM | ≤VI | E | 2002 | LDA | NA | Zambia-Emory HIV Research Project |
| DQ388516 | ZM214M.PL15* | 2 | ZM | ≤VI | E | 2003 | LDA | NA | Zambia-Emory HIV Research Project |
| DQ388517 | ZM233M.PB6* | 2 | ZM | ≤VI | E | 2002 | LDA | NA | Zambia-Emory HIV Research Project |
| DQ411851 | Du151.2* | 2 | ZAkzn | V | E | 1998 | LDA | NA | CAPRISA |
| DQ411852 | Du156.12* | 1B | ZAkzn | ≤IV | E | 1999 | LDA | NA | CAPRISA |
| DQ411853 | Du172.17* | 2 | ZAkzn | VI | E | 1998 | LDA | NA | CAPRISA |
| DQ411854 | Du422.1* | 2 | ZAkzn | V | E | 1998 | LDA | NA | CAPRISA |
| DQ422948 | ZM215F.PB8 | 2 | ZM | ≤VI | E | 2002 | LDA | NA | Zambia-Emory HIV Research Project |
| DQ435682 | CAP45.2.00.G3* | 2 | ZAkzn | IV | I | 2005 | LDA | S | CAPRISA |
| DQ435684 | CAP244.2.00.D3 | 3 | ZAkzn | V | E | 2005 | LDA | NA | CAPRISA |
| FJ443190 | CeCAP210_TA5* | 2 | ZAkzn | I/II | A | 2005 | SGA | S | CAPRISA |
| FJ443274 | CeCAP200_B8a | 1B | ZAkzn | IV | I | 2005 | SGA | S | CAPRISA |
| FJ443316 | CeCAP221_B14 | 2 | ZAkzn | I/II | A | 2006 | SGA | S | CAPRISA |
| FJ443382 | CeCAP177_1A3 | 2 | ZAkzn | I/II | A | 2006 | SGA | S | CAPRISA |
| FJ443533 | Ce703010010_C4 | 2 | MW | II | A | 2006 | SGA | M | CHAVI |
| FJ443575 | Ce703010217_B6 | 2 | MW | V/VI | E | 2007 | SGA | S | CHAVI |
| FJ443639 | Ce703010131_1E2 | 2 | MW | III | I | 2008 | SGA | S | CHAVI |
| FJ443670 | Ce704010083_B8 | 2 | ZAgp | III | I | 2007 | SGA | S | CHAVI |
| FJ443711 | Ce704010069_C6 | 2 | ZAgp | IV | I | 2007 | SGA | S | CHAVI |
| FJ443808 | Ce703010054_2A2 | 2 | MW | V/VI | E | 2007 | SGA | S | CHAVI |
| FJ443841 | CeCAP188_1_D1_14(Rev-) | 2 | ZAkzn | I/II | A | 2007 | SGA | S | CAPRISA |
| FJ443999 | 7030102001E5(Rev-) | 2 | MW | I/II | A | 2007 | SGA | M | CHAVI |
| FJ444017 | Ce703010228_1C4 | 2 | MW | I/II | A | 2007 | SGA | M | CHAVI |
| FJ444059 | 7060101641A7(Rev-) | 2 | ZAnw | I/II | A | 2007 | SGA | S | CHAVI |
| FJ444103 | Ce704809221_1B3 | 2 | ZAgp | I/II | A | 2007 | SGA | S | CHAVI |
| FJ444124 | Ce704810053_2B7 | 2 | ZAgp | I/II | A | 2007 | SGA | S | CHAVI |
| FJ444215 | Ce0393_C3 | 2 | MW | IV | I | 2003 | SGA | S | CHAVI |
| FJ444284 | Ce0626_E6 | 2 | MW | IV | I | 2003 | SGA | S | CHAVI |
| FJ444315 | Ce0665_F2 | 2 | MW | IV | I | 2004 | SGA | S | CHAVI |
| FJ444325 | Ce0682_E4 | 2 | MW | I/II | A | 2003 | SGA | S | CHAVI |
| FJ444366 | Ce0089_G2 | 2 | MW | V | E | 2003 | SGA | S | CHAVI |
| FJ444384 | Ce0985_H7(Rev-) | 2 | MW | I/II | A | 2004 | SGA | S | CHAVI |
| FJ444395 | Ce1086_B2 | 2 | MW | I/II | A | 2004 | SGA | S | CHAVI |
| FJ444421 | Ce1172_H1 | 2 | MW | I/II | A | 2004 | SGA | S | CHAVI |
| FJ444437 | Ce1176_A3 | 2 | MW | I/II | A | 2004 | SGA | S | CHAVI |
| FJ444529 | 1394C9G1(Rev-) | 2 | MW | I/II | A | 2004 | SGA | S | CHAVI |
| FJ444561 | Ce2010_F5 | 3 | MW | IV | I | 2005 | SGA | S | CHAVI |
| FJ444586 | Ce2052_G10 | 2 | MW | I/II | A | 2006 | SGA | S | CHAVI |
| FJ444600 | Ce2060_G9 | 3 | MW | I/II | A | 2005 | SGA | S | CHAVI |
| FJ444612 | Ce2103_E8 | 3 | MW | I/II | A | 2005 | SGA | S | CHAVI |
| FJ496194 | 246F C1 | 2 | ZM | II | A | 2003 | SGA | S | Zambia-Emory HIV Research Project |
| FJ496204 | ZM247v1(Rev-) | 2 | ZM | II | A | 2003 | SGA | S | Zambia-Emory HIV Research Project |
| HM215253 | 0041.v3.c18 | 2 | TZ | V/VI | E | 2004 | SGA | NA | US MHRP |
| HM215254 | 0077.v1.c16 | 2 | TZ | V/VI | E | 2003 | SGA | NA | US MHRP |
| HM215262 | 0921.v2.c14 | 2 | TZ | V/VI | E | 2004 | SGA | NA | US MHRP |
| HM215263 | 0984.v2.c2 | 3 | TZ | V/VI | E | 2004 | SGA | NA | US MHRP |
| HM215278 | 234-F1-16-57 | 2 | TZ | V | E | 2001 | SGA | S | HISIS |
| HM215286 | 304_F2_1_11 | 2 | TZ | V | E | 2001 | SGA | S | HISIS |
| HM215289 | 3168.v4.c10 | 2 | TZ | V/VI | E | 2005 | SGA | NA | US MHRP |
| HM215300 | 3426.v5.c17 | 2 | TZ | 3 months | E | 2005 | SGA | NA | US MHRP |
| HM215302 | 346_F4_D2_12 | 1B | TZ | VI | E | 2001 | SGA | S | HISIS |
| HM215305 | 3637.v5.c3 | 3 | TZ | V/VI | E | 2005 | SGA | NA | US MHRP |
| HM215307 | 3728.v2.c6 | 2 | TZ | III/IV | I | 2004 | SGA | NA | US MHRP |
| HM215308 | 377.v4.c09 | 2 | TZ | 3 months | E | 2004 | SGA | NA | US MHRP |
| HM215311 | 3873.v1.c24 | 2 | TZ | V/VI | E | 2003 | SGA | NA | US MHRP |
| HM215316 | 541-F1_A7_2 | 3 | TZ | 47 days | E | 2001 | SGA | S | HISIS |
| HM215317 | 556_F2_3_25 | 2 | TZ | V | E | 2001 | SGA | M | HISIS |
| HM215318 | 569-F1_37_10 | 2 | TZ | V/VI | E | 2001 | SGA | M | HISIS |
| HM215319 | 6022.v7.c24 | 2 | TZ | 3-9 months | E | 2006 | SGA | NA | US MHRP |
| HM215320 | 6040.v4.c15 | 2 | TZ | V/VI | E | 2005 | SGA | NA | US MHRP |
| HM215324 | 6146.v7.c23 | 2 | TZ | 3-9 months | E | 2006 | SGA | NA | US MHRP |
| HM215326 | 6322.v4.c1 | 2 | TZ | V/VI | E | 2005 | SGA | NA | US MHRP |
| HM215328 | 6471.v1.c16 | 3 | TZ | V/VI | E | 2003 | SGA | NA | US MHRP |
| HM215335 | 6631.v3.c10 | 3 | TZ | V/VI | E | 2004 | SGA | NA | US MHRP |
| HM215336 | 6644.v2.c33 | 1B | TZ | V/VI | E | 2004 | SGA | NA | US MHRP |
| HM215338 | 6785.v5.c14 | 2 | TZ | V/VI | E | 2005 | SGA | NA | US MHRP |
| HM215341 | 6838.v1.c35 | 2 | TZ | I/II | A | 2003 | SGA | NA | US MHRP |
| HM215344 | 6980.v0.c31 | 2 | TZ | I/II | A | 2003 | SGA | NA | US MHRP |
| HM215352 | 933.v4.c4 | 3 | TZ | 3 months | E | 2005 | SGA | NA | US MHRP |
| HM215354 | 98-F4_H5-13 | 3 | TZ | V | E | 2001 | SGA | S | HISIS |
| HM215360 | BF1266.431a | 2 | MW | I/II | A | 2002 | SGA | S | Malawi and HIV-1 in Pregnancy |
| HQ595742 | 1245045 | 2 | ZAkzn | I/II | A | 2008 | SGA | S | SANBS |
| HQ595743 | 19157834_V1 | 2 | ZAmp | I/II | A | 2005 | SGA | S | SANBS |
| HQ595747 | 20721190 | 3 | ZAgp | I/II | A | 2008 | SGA | S | SANBS |
| HQ595748 | 20803520 | 2 | ZAgp | I/II | A | 2008 | SGA | S | SANBS |
| HQ595749 | 20915593 | 2 | ZAgp | I/II | A | 2008 | SGA | S | SANBS |
| HQ595750 | 20927783 | 3 | ZAlp | I/II | A | 2008 | SGA | S | SANBS |
| HQ595751 | 20965238 | 2 | ZAgp | I/II | A | 2009 | SGA | S | SANBS |
| HQ595753 | 21197826_V1 | 3 | ZAnc | I/II | A | 2009 | SGA | S | SANBS |
| HQ595756 | 21283649 | 3 | ZAnc | I/II | A | 2009 | SGA | S | SANBS |
| HQ595757 | 2833264 | 2 | ZAkzn | I/II | A | 2006 | SGA | S | SANBS |
| HQ595759 | 3514597 | 2 | ZAec | III | I | 2007 | SGA | S | SANBS |
| HQ595763 | 20258279_V2 | 2 | ZAmp | IV | I | 2007 | SGA | S | SANBS |
| HQ595766 | 2969249 | 2 | ZAkzn | IV | I | 2007 | SGA | S | SANBS |
| HQ615941 | 21369737_G11_F2 | 3 | ZAkzn | I/II | A | 2009 | SGA | S | SANBS |
| HQ615942 | 3545883_G1_E1 | 2 | ZAec | IV | I | 2007 | SGA | S | SANBS |
| HQ615943 | 20883229_C9_H6 | 3 | ZAgp | I/II | A | 2008 | SGA | S | SANBS |
| HQ615944 | 20198102_E9_G1 | 3 | ZAmp | I/II | A | 2007 | SGA | S | SANBS |
| HQ615945 | 20104663_E11_D2 | 2 | ZAlp | I/II | A | 2007 | SGA | S | SANBS |
| HQ615946 | 21203310_G7_C3 | 2 | ZAgp | I/II | A | 2008 | SGA | S | SANBS |
| HQ615947 | 21492713_B11_E3 | 2 | ZAgp | I/II | A | 2009 | SGA | S | SANBS |
| HQ615948 | 18814602_H8_F3 | 1B | ZAlp | I/II | A | 2005 | SGA | S | SANBS |
| HQ615949 | 21502011_F12_E2 | 2 | ZAgp | I/II | A | 2009 | SGA | S | SANBS |
| HQ615950 | 19715820_A10_H2 | 2 | ZAgp | I/II | A | 2007 | SGA | S | SANBS |
| HQ615951 | 19707346_E8_C6 | 3 | ZAlp | I/II | A | 2006 | SGA | S | SANBS |
| HQ615952 | 2768732_C5_16 | 2 | ZAkzn | I/II | A | 2006 | SGA | S | SANBS |
| HQ615953 | 19252094_A5_G2 | 2 | ZAgp | I/II | A | 2005 | SGA | S | SANBS |
| HQ615954 | 2869751_A4_D3 | 2 | ZAkzn | I/II | A | 2007 | SGA | S | SANBS |
| HQ615955 | 2891391_A2_E1 | 2 | ZAkzn | I/II | A | 2007 | SGA | S | SANBS |
| HQ615956 | 21261106_C12_H2 | 3 | ZAlp | I/II | A | 2009 | SGA | S | SANBS |
| HQ615957 | 21399975_E2_B3 | 2 | ZAgp | IV | I | 2009 | SGA | S | SANBS |
| HQ615958 | 19314479_A2_5 | 2 | ZAnc | I/II | A | 2005 | SGA | S | SANBS |
| HQ615959 | 2759058_F10_B6 | 2 | ZAkzn | I/II | A | 2006 | SGA | S | SANBS |
| HQ615960 | 20286961_C1_H8 | 2 | ZAmp | I/II | A | 2009 | SGA | S | SANBS |
| HQ615961 | 21561324_D3_B5 | 2 | ZAkzn | I/II | A | 2009 | SGA | S | SANBS |
| JN681219 | CA146_H3_3 | 1B | WC | 26 days | E | 2009 | SGA | M | HVTN |
| JN681220 | CA327_D2_2 | 2 | ZAkzn | 71 days | E | 2008 | SGA | M | HVTN |
| JN681221 | CA392_H2_6 | 2 | ZAkzn | I/II | A | 2007 | SGA | S | HVTN |
| JN681222 | CA457_H1_1 | 3 | ZAkzn | 46 days | E | 2008 | SGA | S | HVTN |
| JN681223 | CAP129.1.15_B2_13 | 2 | ZAkzn | IV | I | 2006 | SGA | S | CAPRISA |
| JN681225 | CAP225.1.06_A2_18 | 3 | ZAkzn | III | I | 2005 | SGA | S | CAPRISA |
| JN681226 | CAP237.1.22_B2_2_39 | 3 | ZAkzn | III | I | 2007 | SGA | S | CAPRISA |
| JN681227 | CAP258.2.00_X_23 | 2 | ZAkzn | 49 days | E | 2005 | SGA | M | CAPRISA |
| JN681228 | CAP260.2.00_TA1_1B | 3 | ZAkzn | V | E | 2006 | SGA | M | CAPRISA |
| JN681229 | CAP266.2.00_E9_h6 | 2 | ZAkzn | 47 days | E | 2006 | SGA | M | CAPRISA |
| JN681230 | CAP37.1.18_D2_19 | 1B | ZAkzn | IV | I | 2006 | SGA | M | CAPRISA |
| JN681231 | CAP40.2.01_A3_5 | 2 | ZAkzn | 27 days | E | 2006 | SGA | S | CAPRISA |
| JN681232 | CT072_56_7 | 1B | ZAwc | 21 days | E | 2007 | SGA | S | HVTN |
| JN681233 | CT140_140_B6 | 2 | ZAwc | 14 days | E | 2007 | SGA | S | HVTN |
| JN681234 | CT431_G6_6 | 2 | ZAwc | 20 days | E | 2007 | SGA | S | HVTN |
| JN681236 | CT823_B6_1 | 2 | ZAwc | I/II | A | 2009 | SGA | S | HVTN |
| JN681238 | CT966_E1-7 | 2 | ZAwc | 81 days | E | 2008 | SGA | S | HVTN |
| JN681239 | CT977_69_12 | 2 | ZAwc | 77 days | E | 2008 | SGA | S | HVTN |
| JN681240 | Ko224_T87_2_4 | 2 | ZAnw | 74 days | E | 2008 | SGA | S | HVTN |
| JN681241 | Ko426_T78_10 | 2 | ZAnw | 91 days | E | 2008 | SGA | S | HVTN |
| JN681242 | Ko459_T68_4 | 2 | ZAnw | 91 days | E | 2008 | SGA | S | HVTN |
| JN681243 | Ko756_38_Tb12 | 2 | ZAnw | 77 days | E | 2008 | SGA | S | HVTN |
| JN681244 | Ko870_C2_10 | 2 | ZAnw | 78 days | E | 2008 | SGA | M | HVTN |
| JN681245 | ME067_A10-15 | 2 | ZAgp | 71 days | E | 2008 | SGA | S | HVTN |
| JN681246 | So431_C1_1 | 2 | ZAgp | 90 days | E | 2007 | LDA | S | HVTN |
| JN681247 | So186_H6_5 | 2 | ZAgp | 91 days | E | 2008 | LDA | S | HVTN |
| JN681248 | So405_T24_5 | 2 | ZAgp | 88 days | E | 2008 | LDA | S | HVTN |
| JN681249 | So706_T10b_3 | 2 | ZAgp | 91 days | E | 2008 | SGA | S | HVTN |
| JN681252 | So225_H11_12 | 2 | ZAgp | 49 days | E | 2007 | SGA | S | HVTN |
| JN681253 | CAP290.2.00_23_6 | 2 | ZAkzn | 23 days | E | 2008 | SGA | M | CAPRISA |
| JN681254 | CAP292.2.00_12_4 | 2 | ZAkzn | 27 days | E | 2008 | SGA | S | CAPRISA |
| JN681255 | CAP307.2.00_24_1 | 2 | ZAkzn | 30 days | E | 2008 | SGA | S | CAPRISA |
| JN681256 | CAP310.2.00_20_2 | 2 | ZAkzn | 29 days | E | 2008 | SGA | S | CAPRISA |
| JN681257 | CAP343.2.00_21_2 | 2 | ZAkzn | 52 days | E | 2009 | SGA | S | CAPRISA |
| JN681258 | CAP347.2.00_B1_1 | 2 | ZAkzn | 17 days | E | 2009 | SGA | M | CAPRISA |
| JN967790 | CAP136.1.16_E6_1 | 2 | ZAkzn | V | E | 2006 | SGA | M | CAPRISA |
| JN967791 | CAP174.1.06_F3_1B | 1B | ZAkzn | V | E | 2005 | SGA | S | CAPRISA |
| JN967792 | CAP220.2.00_A8_5B | 2 | ZAkzn | V | E | 2007 | SGA | S | CAPRISA |
| JN967793 | CAP224.1.18_C7_3 | 2 | ZAkzn | V | E | 2006 | SGA | M | CAPRISA |
| JN967794 | CAP269.2.00_F11_1 | 2 | ZAkzn | VI | E | 2006 | SGA | S | CAPRISA |
| JN967797 | CT885_H3_2 | 2 | ZAwc | 38 days | E | 2008 | LDA | S | HVTN |
| JN967798 | 722_G4_16 | 2 | ZAgp | I/II | A | 2007 | LDA | S | HVTN |
| JN977604 | 235080_3G7env2(Rev-) | 1B | ZM | IV | I | 2007 | SGA | S | CHAVI |
| JQ061131 | CT349_39_16 | 1B | ZAwc | 31 days | E | 2007 | SGA | S | HVTN |
| JQ352785 | 20417927.07 | 2 | ZAgp | II | A | 2007 | SGA | S | SANBS |
| JQ352789 | 20355851.2 | 3 | ZAmp | II | A | 2008 | SGA | S | SANBS |
| JQ352790 | 20358510.01 | 2 | ZAmp | II | A | 2008 | SGA | S | SANBS |
| JQ352794 | 1107356.07 | 1B | ZAkzn | III | I | 2008 | SGA | S | SANBS |
| JQ352799 | 1143465.14 | 1B | ZAkzn | II | A | 2008 | SGA | M | SANBS |
| JQ352801 | 1170887.08 | 2 | ZAkzn | II | A | 2008 | SGA | S | SANBS |
| JX131327 | CAP69.1.12_TA7.1 | 2 | ZAkzn | I/II | A | 2006 | SGA | M | CAPRISA |
| KC154012 | 1811_B3.23 | 2 | BW | IV | I | 2004 | SGA | S | Botswana/Harvard Partnership |
| KC154013 | 2865_A11.12 | 2 | BW | III | I | 2005 | SGA | S | Botswana/Harvard Partnership |
| KC154014 | 3312_D6.2 | 2 | BW | III | I | 2005 | SGA | S | Botswana/Harvard Partnership |
| KC154015 | 3603_C11.13 | 2 | BW | I/II | A | 2006 | SGA | S | Botswana/Harvard Partnership |
| KC154016 | CA240_A5.5 | 1B | ZAkzn | 70 days | E | 2008 | SGA | S | HVTN/Phambili |
| KC154017 | CAP301.2.00_C3.20 | 1B | ZAkzn | 4 weeks | E | 2008 | SGA | S | CAPRISA |
| KC154018 | CAP304.2.00_F6.6 | 2 | ZAkzn | 5 weeks | E | 2008 | SGA | S | CAPRISA |
| KC154019 | CAP306.2.00_F9.1 | 2 | ZAkzn | 2 weeks | E | 2008 | SGA | S | CAPRISA |
| KC154020 | CAP308.2.00_E11.35 | 2 | ZAkzn | 10 weeks | E | 2008 | SGA | S | CAPRISA |
| KC154022 | CAP317.2.00_D4.10 | 2 | ZAkzn | 7 weeks | E | 2008 | SGA | S | CAPRISA |
| KC154023 | CAP323.2.00_B6.45 | 2 | ZAkzn | 3.4 weeks | E | 2008 | SGA | S | CAPRISA |
| KC154024 | CAP326.2.00_D9.2 | 2 | ZAkzn | 2 weeks | E | 2008 | SGA | S | CAPRISA |
| KC154025 | CAP330.2.00_F2.41 | 2 | ZAkzn | 2 weeks | E | 2008 | SGA | S | CAPRISA |
| KC154026 | CAP340.2.00_B3.16 | 1B | ZAkzn | 13 weeks | E | 2009 | SGA | S | CAPRISA |
| KC154027 | CAP341.2.00.C10.18 | 2 | ZAkzn | 5 weeks | E | 2009 | SGA | S | CAPRISA |
| KC154028 | CAP382.2.00_D7.19 | 2 | ZAkzn | 7 weeks | E | 2010 | SGA | S | CAPRISA |
| KC156123 | 704010042 | 2 | ZAgp | IV | I | 2003 | SGA | S | CHAVI |
| KC156125 | 705010067 | 3 | ZAkzn | I/II | A | 2003 | SGA | S | CHAVI |
| KC156129 | 705010185 | 2 | ZAkzn | I/II | A | 2003 | SGA | S | CHAVI |
| KC156130 | 705010198 | 2 | ZAkzn | I/II | A | 2003 | SGA | S | CHAVI |
| KC247557 | CH0505.w4.3 | 1A | MW | IV | I | 2008 | SGA | S | CHAVI |
| KF114881 | B005018-8_F6.3 | 2 | BW | III | I | 2006 | SGA | S | Botswana/Harvard Partnership |
| KF114882 | B005582-7_G7.8 | 2 | BW | III | I | 2007 | SGA | S | Botswana/Harvard Partnership |
| KF114884 | CAP291.2.00_H2.15 | 3 | ZAkzn | 11 weeks | E | 2008 | SGA | M | CAPRISA |
| KF114885 | CAP327.2.00_C6.37 | 2 | ZAkzn | 3 weeks | E | 2008 | SGA | S | CAPRISA |
| KF114886 | CAP331.2.00_D7.39 | 2 | ZAkzn | 6 weeks | E | 2009 | SGA | S | CAPRISA |
| KF114887 | CAP332.2.00_C10.1 | 2 | ZAkzn | <10 days | E | 2009 | SGA | S | CAPRISA |
| KF114888 | CAP378.2.00_D2.5 | 2 | ZAkzn | 15 weeks | E | 2010 | SGA | S | CAPRISA |
| KF114889 | CT184_D3.15 | 1B | ZAwc | 9.4 weeks | E | 2007 | SGA | S | HVTN |
| KF114890 | CT565_C7.48 | 3 | ZAwc | 51 days | E | 2009 | SGA | S | HVTN |
| KF114891 | CT810_G4-7 | 2 | ZAwc | 5 weeks | E | 2007 | SGA | S | HVTN |
| KF114892 | Ko243_H6.3 | 3 | ZAnw | 58 days | E | 2009 | SGA | S | HVTN |
| KF114893 | Me178_G6.16 | 3 | ZAgp | 64 days | E | 2008 | SGA | S | HVTN |
| KF114894 | SO032_A2.8-1 | 1A | ZAgp | V/VI | E | 2008 | SGA | S | HVTN |
| KF114895 | SO607_B6.9 | 3 | ZAgp | 53 days | E | 2008 | SGA | S | HVTN |
| KJ598853 | CAP363.2.00_10_3 | 2 | ZAkzn | 37 days | E | 2009 | SGA | S | CAPRISA |
| KJ700457 | CAP206.1.B5 | 2 | ZAkzn | 4 weeks | E | 2005 | SGA | S | CAPRISA |
| KJ700458 | CH0694.ENV | 2 | MW | III | I | 2008 | SGA | S | CHAVI |

^a^ Country and provincial origin: Botswana (BW); Malawi (MW); Tanzania (TZ); Zambia (ZM); South Africa Western Cape Province (ZAwc); ZA Eastern Cape Province (ZAec); ZA North West Province (ZAnw); ZA Kwazulu-Natal (ZAkzn); ZA Western Mpumalanga Cape (ZAmp); ZA Western Northern Cape (ZAnc); ZA Limpopo Province (ZAlp); ZA Gauteng Province (ZAgp).

^b^TF is transmitted founder and nTF is non-transmitted founder.

^c^ Infection Staging: A (Acute) is Fiebig Stage I/II; I (Intermediate) is Fiebig stage III/IV; and E (Early) is Fiebig stage V/VI. Where Fiebig staging were unavailable, samples were assigned E. All samples were collected with 100 days of a previous HIV-1 negative diagnosis.

^d^SGA is Single Genome Amplification; LDA is Limiting Dilution Amplification

^e^ Multiplicity of infection , multiple (M), single (S) or NA (not available).

^f^ South African National Blood Service (SANBS); Center for HIV/AIDS Vaccine Immunology (CHAVI); HIV Vaccines Trials Network (HVTN); Centre for the Aids Programme of Research in South Africa (CAPRISA); HIV super infection study (HISIS); US Military HIV Research Program (US MHRP);

* A total of 12 pseudoviruses that were included in a previous study, first describing tiered categorization of pseudoviruses are indicated (16).
