# Supplementary material for: Features of Recently Transmitted HIV-1 Clade C Viruses that Impact Antibody Recognition: Implications for Active and Passive Immunization
Source: PLoS Pathog. 2016 Jul 19;12(7):e1005742. doi: 10.1371/journal.ppat.1005742 (PMC4951126; doi:10.1371/journal.ppat.1005742)
Supplement: S2 Table — (DOCX) [file ppat.1005742.s010.docx]

**TABLE S2** Pre-screening of 54 of chronic serum samples against a panel of seven pseudoviruses, including 4 clade C’s (CAP8.6F, CAP255.16, Du156.12 and a clade C consensus); 2 clade B’s (6535 and a clade B consensus); and 1 clade A (Q23.17)

|  | ***Clade C Viruses*** | | | | ***Clade B Viruses*** | | ***Clade A Viruses*** |  |  |
| --- | --- | --- | --- | --- | --- | --- | --- | --- | --- |
| ***Patient ID*** | ***ConC*** | ***CAP8.6F*** | ***CAP255.16*** | ***DU156.12*** | ***ConB*** | ***6535*** | ***Q23.17*** | ***% Neutralized*** | ***Selected for Screening*** |
| CAP287 | 2836 | 77 | 685 | 452 | 414 | 114 | 641 | 100% | Y |
| CAP292 | 559 | 47 | 88 | 84 | 190 | 561 | 65 | 100% | Y |
| CAP309 | 547 | 67 | 77 | 238 | 231 | 224 | 46 | 100% |  |
| CAP370 | 2914 | 130 | 52 | 332 | 577 | 1892 | 2786 | 100% |  |
| CT02 | 4495 | 1430 | 860 | 714 | 3368 | 3666 | 1940 | 100% | Y |
| CT07 | 996 | 563 | 417 | 337 | 266 | 305 | 310 | 100% | Y |
| PHRU_01 | 3859 | 142 | 1504 | 565 | 1360 | 1806 | 1016 | 100% | Y |
| PHRU_17 | 3469 | 6052 | 255 | 339 | 215 | 332 | 1118 | 100% | Y |
| PHRU_20 | 2666 | 72 | 365 | 101 | 376 | 321 | 5936 | 100% | Y |
| PHRU_21 | 938 | 83 | 56 | 254 | 97 | 2831 | 139 | 100% | Y |
| PHRU_23 | 254 | 60 | 423 | 232 | 331 | 790 | 64 | 100% | Y |
| CAP288 | 159 | 77 | 107 | 81 | 41 | 80 | <40 | 86% | Y |
| CAP315 | 299 | <40 | 67 | 96 | 818 | 109 | 156 | 86% | Y |
| CAP341 | 1582 | <40 | 449 | 390 | 722 | 782 | 3985 | 86% |  |
| CAP363 | 1003 | <40 | 62 | 57 | 72 | 79 | 1028 | 86% | Y |
| CT11 | 239 | 85 | 64 | 55 | <40 | 94 | 63 | 86% | Y |
| CT19 | 4493 | <40 | 830 | 153 | 1074 | 71 | 634 | 86% | Y |
| PHRU_28 | 12218 | <40 | 567 | 648 | 2899 | 358 | 1413 | 86% | Y |
| CAP318 | 193 | 47 | 3435 | 177 | <40 | <40 | 281 | 71% | Y |
| CT14 | 1423 | <40 | 339 | 116 | 566 | 941 | <40 | 71% | Y |
| CT15 | 10210 | 3013 | 80 | 190 | <40 | <40 | 1526 | 71% | Y |
| PHRU_16 | 2398 | <40 | 579 | 871 | 539 | 289 | <40 | 71% | Y |
| CT01 | 433 | <40 | 66 | 302 | <40 | <40 | 459 | 57% |  |
| CT12 | 865 | <40 | 2543 | 213 | 121 | <40 | <40 | 57% | Y |
| CT18 | 4993 | <40 | 205 | <40 | 738 | <40 | 380 | 57% | Y |
| PHRU_24 | 44 | 181 | 107 | <40 | 65 | <40 | <40 | 57% |  |
| CAP302 | 51 | 67 | <40 | 223 | <40 | <40 | <40 | 43% | Y |
| CAP349 | 6433 | <40 | <40 | 42 | 276 | <40 | <40 | 43% | Y |
| CT06 | 917 | <40 | <40 | 106 | <40 | <40 | 93 | 43% |  |
| CT20 | 11204 | <40 | 60 | 44 | <40 | <40 | <40 | 43% | Y |
| PHRU_07 | 2168 | <40 | 147 | <40 | <40 | <40 | 409 | 43% | Y |
| PHRU_09 | 530 | <40 | <40 | 288 | 204 | <40 | <40 | 43% | Y |
| PHRU_11 | 76 | 425 | <40 | 42 | <40 | <40 | <40 | 43% | Y |
| CAP334 | 4955 | <40 | <40 | 79 | <40 | <40 | <40 | 29% | Y |
| CAP354 | <40 | <40 | <40 | 42 | <40 | <40 | 77 | 29% | Y |
| CT04 | 108 | <40 | 90 | <40 | <40 | <40 | <40 | 29% |  |
| CT16 | 101 | <40 | 58 | <40 | <40 | <40 | <40 | 29% |  |
| PHRU_08 | 60 | <40 | <40 | 83 | <40 | <40 | <40 | 29% |  |
| PHRU_18 | 106 | <40 | <40 | 142 | <40 | <40 | <40 | 29% |  |
| CAP342 | 55 | <40 | <40 | <40 | <40 | <40 | <40 | 14% |  |
| CT08 | <40 | <40 | 99 | <40 | <40 | <40 | <40 | 14% |  |
| CT09 | <40 | <40 | 502 | <40 | <40 | <40 | <40 | 14% | Y |
| CT10 | <40 | <40 | <40 | 58 | <40 | <40 | <40 | 14% |  |
| PHRU_22 | 44 | <40 | <40 | <40 | <40 | <40 | <40 | 14% |  |
| CAP338 | <40 | <40 | <40 | <40 | <40 | <40 | <40 | 0% |  |
| CAP372 | <40 | <40 | <40 | <40 | <40 | <40 | <40 | 0% |  |
| CT03 | <40 | <40 | <40 | <40 | <40 | <40 | <40 | 0% |  |
| CT05 | <40 | <40 | <40 | <40 | <40 | <40 | <40 | 0% |  |
| CT13 | <40 | <40 | <40 | <40 | <40 | <40 | <40 | 0% |  |
| CT17 | <40 | <40 | <40 | <40 | <40 | <40 | <40 | 0% |  |
| PHRU_03 | <40 | <40 | <40 | <40 | <40 | <40 | <40 | 0% |  |
| PHRU_06 | <40 | <40 | <40 | <40 | <40 | <40 | <40 | 0% |  |
| PHRU_12 | <40 | <40 | <40 | <40 | <40 | <40 | <40 | 0% |  |
| PHRU_14 | <40 | <40 | <40 | <40 | <40 | <40 | <40 | 0% |  |
