# Supplementary material for: Features of Recently Transmitted HIV-1 Clade C Viruses that Impact Antibody Recognition: Implications for Active and Passive Immunization
Source: PLoS Pathog. 2016 Jul 19;12(7):e1005742. doi: 10.1371/journal.ppat.1005742 (PMC4951126; doi:10.1371/journal.ppat.1005742)
Supplement: S3 Table — (DOCX) [file ppat.1005742.s011.docx]

**TABLE S3** Demographic characteristics of the subset of thirty serum and plasma samples selected based on evidence for cross-reactivity, sufficient sample availability, and geographical representation

|  |  | ***Collection date*** | ***Estimated Infection date^a^*** | ***dpi*** | ***Gender*** | ***Diagnosis Date*** | ***CD4 count*** | ***Viral load (cp/ml)*** |
| --- | --- | --- | --- | --- | --- | --- | --- | --- |
| ***Durban Plasma*** | CAP287 | 16-Apr-12 | 01-Dec-07 | 1598 | F | NA | 326 | 17825 |
|  | CAP288 | 24-May-11 | 25-Jan-08 | 1215 | F | NA | 312 | 4637 |
|  | CAP292 | 27-Sep-11 | 01-Mar-08 | 1305 | F | NA | 392 | 32336 |
|  | CAP302 | 09-Jun-11 | 07-May-08 | 1128 | F | NA | 211 | 93071 |
|  | CAP315 | 05-Dec-11 | 27-Aug-08 | 1195 | F | NA | 390 | 17056 |
|  | CAP318 | 27-Mar-12 | 02-Sep-08 | 1302 | F | NA | 347 | 76663 |
|  | CAP334 | 29-Mar-12 | 27-Nov-08 | 1218 | F | NA | 352 | 9525 |
|  | CAP349 | 25-Apr-12 | 25-Feb-09 | 1155 | F | NA | 358 | 99010 |
|  | CAP354 | 18-Sep-12 | 07-May-09 | 1230 | F | NA | 378 | 31509 |
|  | CAP363 | 20-Aug-12 | 06-Jul-09 | 1141 | F | NA | 365 | 8468 |
| ***Cape Town Serum*** | CT02 | 22-Jul-13 | NA | NA | F | 2006 | 137 | 725694 |
|  | CT07 | 30-Jul-13 | NA | NA | M | 2000 | 327 | 18705 |
|  | CT09 | 30-Jul-13 | NA | NA | F | 2012 | 333 | 66771 |
|  | CT11 | 01-Aug-13 | NA | NA | F | 2006 | 301 | 420229 |
|  | CT12 | 02-Aug-13 | NA | NA | F | 2013 | 216 | 46603 |
|  | CT14 | 06-Aug-13 | NA | NA | F | 08-Jul-13 | 247 | 30144 |
|  | CT15 | 08-Aug-13 | NA | NA | F | 2012 | 337 | 38665 |
|  | CT18 | 16-Aug-13 | NA | NA | F | 2009 | 303 | 336805 |
|  | CT19 | 20-Aug-13 | NA | NA | F | 15-Jul-13 | 215 | 43189 |
|  | CT20 | 20-Aug-13 | NA | NA | F | Mar-10 | 440 | 13329 |
| ***Soweto Serum*** | PHRU_01 | 16-Sep-13 | NA | NA | F | Jan-04 | 398 | 4 352 |
|  | PHRU_07 | 10-Sep-13 | NA | NA | F | Apr-07 | 369 | 1 947 |
|  | PHRU_09 | 13-Sep-13 | NA | NA | F | 11-Nov-10 | 238 | 26 034 |
|  | PHRU_11 | 13-Sep-13 | NA | NA | F | Apr-04 | 213 | 67 301 |
|  | PHRU_16 | 10_Sep13 | NA | NA | F | 29-Feb-08 | 281 | 198 481 |
|  | PHRU_17 | 25-Sep-13 | NA | NA | F | 29-Sep-04 | 335 | 128 243 |
|  | PHRU_20 | 13-Sep-13 | NA | NA | F | Aug-08 | 232 | 28 097 |
|  | PHRU_21 | 18-Sep-13 | NA | NA | F | Jan-10 | 413 | 9 727 |
|  | PHRU_23 | 30-Sep-13 | NA | NA | F | Jun-06 | 366 | 95 847 |
|  | PHRU_28 | 24-Oct-13 | NA | NA | F | Nov-11 | 381 | 129 135 |

^a^ Estimated date of infection was determined as the midpoint between the last RNA negative sample and the first Seropositive sample.
