# Supplementary material for: Features of Recently Transmitted HIV-1 Clade C Viruses that Impact Antibody Recognition: Implications for Active and Passive Immunization
Source: PLoS Pathog. 2016 Jul 19;12(7):e1005742. doi: 10.1371/journal.ppat.1005742 (PMC4951126; doi:10.1371/journal.ppat.1005742)
Supplement: S4 Table — (DOCX) [file ppat.1005742.s012.docx]

**TABLE S4** Co-receptor usage predictions and *in vitro* Trofile assay confirmation

|  |  | ***Predictions*** | | ***In Vitro*** |
| --- | --- | --- | --- | --- |
| ***Accession Number*** | ***Clone Name*** | ***Geno2pheno*** | ***WebPSSM*** | ***Trofile*** |
| FJ443808 | Ce703010054_2A2 | X4 | X4 | R 5 |
| HM215318 | 569-F1_37_10 | X4 | X4 | R 5 |
| HQ595756 | 21283649 | X4 | X4 | R 5 |
| HQ615952 | 2768732_C5_16 | X4 | R 5 | R 5 |
| JN681226 | CAP237.1.22_B2_2_39 | X4 | X4 | R 5 |
| JN967797 | CT885_H3_2 | X4 | X4 | R 5 |
| JQ352789 | 20355851.2 | X4 | X4 | R 5 |
| KC154028 | CAP382.2.00_D7.19 | X4 | X4 | R 5 |
